# Diagnosing mild traumatic brain injury using saliva RNA compared to cognitive and balance testing

**DOI:** 10.1002/ctm2.197

**Published:** 2020-10-04

**Authors:** Steven D. Hicks, Cayce Onks, Raymond Y. Kim, Kevin J. Zhen, Jayson Loeffert, Andrea C. Loeffert, Robert P. Olympia, Gregory Fedorchak, Samantha DeVita, Aakanksha Rangnekar, John Leddy, Mohammad N. Haider, Zofia Gagnon, Callan D. McLoughlin, Matthew Badia, Jason Randall, Miguel Madeira, Aaron M. Yengo‐Kahn, Justin Wenzel, Matthew Heller, Hallie Zwibel, Aaron Roberts, Samantha Johnson, Chuck Monteith, Michael N. Dretsch, Thomas R. Campbell, Rebekah Mannix, Christopher Neville, Frank Middleton

**Affiliations:** ^1^ Department of Pediatrics Penn State College of Medicine Hershey Pennsylvania; ^2^ Department of Family Medicine Penn State College of Medicine Hershey Pennsylvania; ^3^ Department of Orthopedics and Rehabilitation Penn State College of Medicine Hershey Pennsylvania; ^4^ Department of Emergency Medicine Penn State College of Medicine Hershey Pennsylvania; ^5^ Quadrant Biosciences Syracuse New York; ^6^ UBMD Orthopedics and Sports Medicine, Jacobs School of Medicine and Biomedical Sciences State University of New York Buffalo New York; ^7^ Department of Biomedical Science Marist College Poughkeepsie New York; ^8^ Department of Environmental Science School of Science Marist College Poughkeepsie New York; ^9^ Department of Biology, School of Science Marist College Poughkeepsie New York; ^10^ Vanderbilt Sports Concussion Center Vanderbilt University Medical Center Nashville Tennessee; ^11^ Department of Family Medicine New York Institute of Technology College of Osteopathic Medicine Old Westbury New York; ^12^ Adena Bone and Joint Center Adena Regional Medical Center Chillicothe Ohio; ^13^ Athletic Training Department Colgate University Hamilton New York; ^14^ US Army Medical Research Directorate‐West Walter Reed Army Institute of Research Joint Base Lewis–McChord Washington; ^15^ Athletic Training Department Old Dominion University Norfolk Virginia; ^16^ Division of Emergency Medicine, Boston Children's Hospital Harvard Medical School Boston Massachusetts; ^17^ Department of PT Education, Orthopedics, and Neuroscience SUNY Upstate Medical University Syracuse New York; ^18^ Department of Neuroscience and Physiology SUNY Upstate Medical University Syracuse New York

**Keywords:** balance, biomarker, concussion, diagnosis, neurocognition, RNA, saliva, traumatic brain injury

## Abstract

**Background:**

Early, accurate diagnosis of mild traumatic brain injury (mTBI) can improve clinical outcomes for patients, but mTBI remains difficult to diagnose because of reliance on subjective symptom reports. An objective biomarker could increase diagnostic accuracy and improve clinical outcomes. The aim of this study was to assess the ability of salivary noncoding RNA (ncRNA) to serve as a diagnostic adjunct to current clinical tools. We hypothesized that saliva ncRNA levels would demonstrate comparable accuracy for identifying mTBI as measures of symptom burden, neurocognition, and balance.

**Methods:**

This case‐control study involved 538 individuals. Participants included 251 individuals with mTBI, enrolled ≤14 days postinjury, from 11 clinical sites. Saliva samples (n = 679) were collected at five time points (≤3, 4‐7, 8‐14, 15‐30, and 31‐60 days post‐mTBI). Levels of ncRNAs (microRNAs, small nucleolar RNAs, and piwi‐interacting RNAs) were quantified within each sample using RNA sequencing. The first sample from each mTBI participant was compared to saliva samples from 287 controls. Samples were divided into testing (n = 430; mTBI = 201 and control = 239) and training sets (n = 108; mTBI = 50 and control = 58). The test set was used to identify ncRNA diagnostic candidates and create a diagnostic model. Model accuracy was assessed in the naïve test set.

**Results:**

A model utilizing seven ncRNA ratios, along with participant age and chronic headache status, differentiated mTBI and control participants with a cross‐validated area under the curve (AUC) of .857 in the training set (95% CI, .816‐.903) and .823 in the naïve test set. In a subset of participants (n = 321; mTBI = 176 and control = 145) assessed for symptom burden (Post‐Concussion Symptom Scale), as well as neurocognition and balance (ClearEdge System), these clinical measures yielded cross‐validated AUC of .835 (95% CI, .782‐.880) and .853 (95% CI, .803‐.899), respectively. A model employing symptom burden and four neurocognitive measures identified mTBI participants with similar AUC (.888; CI, .845‐.925) as symptom burden and four ncRNAs (.932; 95% CI, .890‐.965).

**Conclusion:**

Salivary ncRNA levels represent a noninvasive, biologic measure that can aid objective, accurate diagnosis of mTBI.

## Graphical abstract

Levels of nine salivary noncoding RNAs (ncRNAs) accurately differentiated 251 individuals with mild traumatic brain injury (mTBI) from 287 peers with mTBI‐like symptoms.

ncRNA performance (area under the curve [AUC] = .857) was similar to other diagnostic adjuncts: neurocognition (AUC = .835) and balance (AUC = .853).

Combining ncRNA levels with symptom severity (AUC = .932) demonstrated comparable accuracy to current clinical guidelines (symptom severity plus neurocognition; AUC = .888).

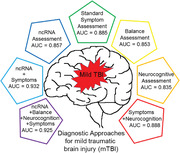

## 1 INTRODUCTION

Mild traumatic brain injury (mTBI) is characterized by brief confusion, loss of consciousness, posttraumatic amnesia, and/or other transient neurological abnormalities (eg, seizure), with a Glasgow Coma Scale score of 13‐15 after 30 min postinjury or later.^1^ Nearly 3 million mTBIs occur in the United States each year, and the majority occur in adolescents and young adults.^2^, ^3^, ^4^ The prevalence of mTBI in adolescents is on the rise, resulting in increasing economic and healthcare system burden.^5^, ^6^

mTBI is associated with significant morbidity, including headaches, fatigue, and difficulties with concentration.^2^, ^7^ mTBI is also associated with missed school or work, and increased healthcare utilization.^8^ mTBI can have a wide range of effects on physical, cognitive, and psychological function, negatively impacting cognitive abilities, academic performance, behavior, social interaction, and employment.^9^, ^10^, ^11^ It can be difficult to identify the physical and neurocognitive effects of mTBI, as some effects may be attributed to other causes such as anxiety, depression, attention deficit hyperactivity disorder (ADHD), exercise‐related fatigue, or chronic headache disorder. Because of this, physical and neurocognitive measures have limited specificity and utility for diagnostic purposes when administered alone.^12^ As such, identifying reliable objective biomarkers is necessary to effectively screen, diagnose, and treat mTBI.

Another obstacle when screening for mTBI is that symptoms and deficits often have delayed emergence.^10^ This temporal pattern can be attributed to the natural progression in pathophysiologic changes, underdiagnosis, and/or underreporting.^12^, ^13^ As early diagnosis and intervention can minimize latent adverse events (such as persistent symptoms) and improve one's quality of life, the ability to quickly and accurately diagnose mTBI is critical to improving outcomes.^5^, ^13^ However, studies show that mTBI is both underdiagnosed and underreported.^14^, ^15^

The 2018 guidelines on the diagnosis of mTBI from the Centers for Disease Control and Prevention (CDC) advise healthcare professionals to use age‐appropriate, validated symptom rating scales as a component of mTBI evaluation (moderate, level B evidence), which can be accompanied by computerized cognitive testing (moderate, level C evidence).^16^ The guidelines specifically state that healthcare professionals should not use biomarkers outside of a research setting (high, level R evidence), and the utility of balance testing for mTBI *diagnosis* is not discussed. Unfortunately, individuals seeking to expedite or delay return‐to‐activities may manipulate cognitive measures by “sandbagging” baseline tests, or exaggerate postinjury symptom reports.^17^, ^18^, ^19^ In addition, the signs and symptoms of mTBI can be subtle and easily missed in acute care settings.^14^ To add to that challenge, many acute care and emergency medicine providers report insufficient training to effectively diagnose and manage mTBI.^20^ Thus, a reliable and objective mTBI diagnosis remains a critical unmet need.

A number of alternative technologies for traumatic brain injury (TBI) diagnosis have been proposed, including neuroimaging, electrophysiology, and blood biomarkers.^21^, ^22^ Though these approaches have shown promise, they also carry limitations that may impede clinical adoption.^23^ For example, neuroimaging and electrophysiology technologies require expensive, nonportable equipment and specialist interpretation. Changes in blood‐based proteins and lipids following TBI demonstrate utility for determining risk of intracranial bleeding and the utility of computed tomography in adults.^24^ Recent advances in blood‐based biomarkers suggest they may soon provide clinical utility for mTBI diagnosis.^25^, ^26^, ^27^ Yet, most mTBIs do not result in intracranial bleeding. Further, protein biomarkers are typically present at low concentrations (fM to pM), are susceptible to degradation, and may have difficulty crossing the blood‐brain barrier in cases of mild injury.^28^, ^29^, ^30^ Further, athletic trainers who lack venipuncture training cannot employ blood‐based biomarkers field‐side, where over a third of mTBIs in adolescents and young adults occur.^3^, ^10^

Micro‐ribonucleic acids (miRNAs) are a class of small, noncoding RNAs (ncRNAs) that regulate protein transcription through sequence‐specific binding and degradation of messenger RNA. Like other ncRNAs, including small nucleolar RNAs (snoRNAs) and piwi‐interacting RNAs (piRNAs), miRNAs play an important role in brain development.^31^ They have also been implicated in both severe and mild forms of TBI.^32^, ^33^ Neurons can package ncRNAs within protective vesicles, allowing them to traverse the extracellular space, dock at distant cells, and influence gene expression.^34^ Thus, measurement of salivary ncRNAs arising from the five cranial nerves in the oropharynx may provide a noninvasive molecular window into the physiology of mTBI.^35^, ^36^ Previously, we showed that miRNA levels in cerebrospinal fluid are mirrored in the saliva of pediatric patients with mTBI.^37^ In a follow‐up study of 52 children with mTBI, saliva miRNA levels accurately predicted duration of symptoms.^38^ Finally, we showed that miRNA levels change within hours of mTBI, and this response occurs in saliva before serum.^39^

To date, the diagnostic potential of salivary ncRNAs has not been assessed in a large mTBI cohort with adequate controls (ie, individuals with orthopedic injury [OI], chronic headache, recent exercise, and neuropsychological comorbidities). Further, the diagnostic accuracy of ncRNAs relative to current standard‐of‐care assessments has not been tested. We hypothesized that measurement of salivary ncRNAs would demonstrate comparable accuracy for discerning mTBI in children and young adults, relative to symptom burden, neurocognitive assessment, or balance measures. We also posited that a composite assessment, combining saliva ncRNA measures with other objective tools and subjective symptom reports, would increase diagnostic accuracy. These hypotheses were tested in a multisite, case‐control study, involving 538 children and young adults (251 with mTBI and 287 controls).

## 2 MATERIALS AND METHODS

Ethical approval for this study was provided through a central institutional review board (Western IRB 1271583). Institutional approval was also provided by independent institutional review boards at the Penn State College of Medicine (STUDY00003729), SUNY Upstate Medical University (1070727), Marist College (#S18‐033), SUNY Buffalo (study #00004347), Vanderbilt University Medical Center (#181814), and Department of the Army, Regional Health Command‐Atlantic (#1510001‐1). Written, informed consent was obtained for all participants. Written assent was provided by participants under 18 years of age. The study was registered in the clinicaltrials.gov registry (NCT02901821).

### 2.1 Participants

This prospective multicenter, case‐control study included a convenience sample of 538 individuals, ages 5‐66 years. There were 251 individuals with a clinical diagnosis of mTBI, defined by the 2016 Concussion in Sport Group criteria as rapid‐onset, short‐lived, spontaneously resolving impairment in neurologic function, typically reflected by functional disturbance rather than structural injury, and characterized by a range of clinical symptoms (eg, headache, dizziness, confusion, and amnesia) that may or may not involve loss of consciousness.^40^ This broad definition of mTBI was chosen to include “mild” cases that did not result in loss of consciousness, and potentially improve the sensitivity of a resulting ncRNA diagnostic algorithm in future investigations. The mTBI group included individuals with sport‐related and nonsport mechanisms of injury, enrolled from emergency departments (EDs), sports medicine clinics, urgent care centers, concussion specialty clinics, and outpatient primary care clinics at initial clinical presentation (within 14 days of injury). Following enrollment, saliva and survey data were collected from mTBI participants across five time points: <72 h (n = 129), 4‐7 days (n = 120), 8‐14 days (n = 190), 15‐30 days (n = 105), and 31‐60 days postinjury (n = 135). The first sample from each participant was used to generate the diagnostic algorithm, whereas longitudinal samples were used only to explore the physiologic characteristics of ncRNA candidates postinjury (see Section 2.8, below).

The mTBI group was compared to a control group of 287 individuals with absence of mTBI in the previous 12 weeks and clinical resolution of any previous mTBI. The control group was enrolled from outpatient primary care clinics, emergency departments, outpatient specialty care clinics, and sports medicine clinics. To provide comparable rates of OI, recent exercise (within 60 min of sample collection), and neuropsychological conditions (eg, depression and anxiety) between control and mTBI groups, 25 control participants were excluded from downstream analysis (Figure S1). OI was defined as an upper/lower extremity sprain, contusion, or fracture within 14 days of enrollment. Recent exercise was defined as ≥30 min of mild/moderate physical activity on the day of enrollment. A subset of control participants with recent exercise (25/38) included collegiate football athletes, for whom head impacts were recorded. Research staff directly observed and recorded helmet‐to‐ground, helmet‐to‐helmet, and helmet‐to‐body impacts for each player during a full‐contact practice immediately prior to saliva collection. None of the football athletes were diagnosed with mTBI by athletic training staff in the course of the practice.

All participants were enrolled from April 2017 through February 2020 at 11 institutions: Adena Health System (Chillicothe, OH; n = 37), Bridgewater College (Bridgewater, VA; n = 20), Colgate University (Hamilton, NY; n = 108), the United States Army (Fort Benning, GA; n = 15), Marist College (Poughkeepsie, NY; n = 25), Penn State College of Medicine (Hershey, PA; n = 224), New York Institute of Technology (Old Westbury, NY; n = 36), State University of New York (SUNY) at Buffalo Jacobs School of Medicine (Buffalo, NY; n = 11), SUNY Upstate Medical University (Syracuse, NY; n = 38), Temple University (Philadelphia, PA; n = 7), and Vanderbilt University Medical Center (Nashville, TN; n = 17).

Exclusion criteria for all participants were primary language other than English, pregnancy, active periodontal disease, neurologic disorder (eg, epilepsy, multiple sclerosis, and hydrocephalus), drug or alcohol dependency, current upper respiratory infection, legally appointed guardian, or inability to provide consent/assent due to intellectual disability. Participants were excluded from the mTBI group for Glasgow Coma Score (GCS) ≤12 at the time of initial injury, penetrating head injury, symptoms attributable to underlying psychological disorder (eg, depression, anxiety, and posttraumatic stress disorder), overnight hospitalization for current mTBI, presentation for clinical care >14 days after the initial injury, skull fracture, or findings of intracranial bleed on CT or MRI (if performed). The proportion of participants who underwent intracranial imaging was not recorded, but anecdotally, the majority of mTBI participants had no imaging performed. Participants were excluded from the control group for ongoing rheumatologic or neoplastic condition, mTBI in the previous 90 days, or persistence of symptoms from a previous mTBI.

Participants were divided into a training set (80% of samples; n = 430 [mTBI = 201 and controls = 229]) used for ncRNA exploration and creation of predictive algorithms, and a naïve testing set (20% of samples; n = 108 [mTBI = 50 and controls = 58]) used only to validate the accuracy of predictive algorithms. Samples were assigned randomly to training and testing sets to ensure equal representation of age, sex, mTBI status, and symptom severity across cohorts. Only one sample from each participant was used. For mTBI participants, the first sample collected postinjury was employed.

### 2.2 Demographic and medical data collection

Participant characteristics were collected via survey, administered by research staff in the clinical care setting. For children ≤12 years of age, parents assisted with survey completion. When possible, survey responses were confirmed through review of the electronic medical record. For all participants, the following medical and demographic characteristics were collected: age (years), sex (male/female), ethnicity (White, Black or African American, Asian, Hispanic or Latino, American Indian or Alaskan Native, and Native Hawaiian or Pacific Islander), weight (kg), height (cm), body mass index (kg/m^2^), dietary restrictions (presence/absence), and chronic medical issues (presence/absence of ADHD, anxiety, depression, and chronic headache disorder). All participants reported presence/absence of any previous TBI, time since most recent TBI (days), and number of previous TBIs. Participants in the mTBI group reported presence/absence of loss of consciousness, and antero‐ or retro‐grade memory loss associated with recent mTBI. All participants reported presence/absence of current OI.

### 2.3 Symptom assessment

For a subset of all participants (n = 387; mTBI = 208 and control = 179), 22 symptoms were self‐reported on a 7‐point Likert scale using the Post‐Concussion Symptom Scale (PCSS).^41^ Total symptom severity (sum of all Likert scores) and total symptom burden (sum of all symptoms >0) were calculated for each participant. Presence or absence of symptoms >3 weeks after initial injury was assessed for mTBI participants through self‐report on the PCSS, or through electronic medical record review (where available).

### 2.4 Balance assessment

Balance was also assessed for a portion of participants (n = 321; mTBI = 176 and control = 145) using the validated ClearEdge system^42^, ^43^ (Quadrant Biosciences Inc, Syracuse, NY), as we have previously reported.^39^ ClearEdge uses an inertial sensor (worn at the L4 vertebral level) to measure body sway in three dimensions during eight different stances: two legs eyes open (TLEO), tandem stance eyes open (TSEO), two legs eyes closed (TLEC), tandem stance eyes closed (TSEC), two legs eyes open on a foam pad (TLEOFP), two legs eyes closed on a foam pad (TLECFP), tandem stance eyes open on a foam pad (TSEOFP), and tandem stance eyes closed on a foam pad (TSECFP). The eight balance tests were scored in terms of power spectral density of acceleration, scaled from 0 (poor balance) to 100 (superior balance). There were 21 participants who were unable to complete balance testing due to OI, or extreme postural instability that posed an increased risk for falling.

### 2.5 Neurocognitive assessment

Computerized neurocognitive assessment was performed in the same subset of participants (n = 321) using the following tests: simple reaction time (SRT1) in which the participant recognizes the presence of an object on the screen and taps it, procedural reaction time (PRT) in which the participant recognizes one of four numbers and taps one of two buttons, go/no‐go (GNG) in which the participant recognizes a green or gray object and only taps in response to a gray, and a repeat of the simple reaction time test (SRT2). This battery of tests is part of the Defense Automated Neurobehavioral Assessment (DANA).^44^, ^45^ Though a more extensive neurocognitive battery may demonstrate improved detection of mTBI, these four tests were selected to reduce participant dropout rates, and promote generalizability within busy clinical settings. The four cognitive tests were scored for speed and accuracy using a mean throughput measure of mental efficiency, calculated as the mean number of correct responses per minute for each test. Mean‐throughput is not a scaled score and is test dependent, with higher scores reflecting better performance (eg, faster reaction time). All individual scores were objectively calculated with computerized software.

### 2.6 Salivary ncRNA assessment

Saliva was collected from all participants (n = 538) in a nonfasting state, following a tap water rinse. Highly absorbent OraCollect Swabs (DNA Genotek, Ottawa Canada) were applied under the tongue and near the parotid glands bilaterally for 10‐15 s. Swabs were submerged in nucleic acid stabilizing solution and stored at room temperature, prior to shipping via priority mail to the Molecular Analysis Core Facility at SUNY Upstate Medical University. RNA was isolated from each saliva sample using the miRNeasy Kit (Qiagen, Inc, Germantown, MD) according to the manufacturer's instructions, as we have previously described.^39^ RNA quality was assessed using the Agilent Technologies Bioanalyzer on the RNA Nanochip. RNA‐sequencing libraries were prepared using the TruSeq Stranded Small RNA Kit (Illumina) according to manufacturer instructions. Samples were indexed in batches of 48, with a targeted sequencing depth of 10 million reads per sample. Sequencing was performed using 50 base pair, single‐end reads, using an Illumina NextSeq 500 instrument. Fastq files were trimmed to remove adapter sequences using Cutadapt version 1.2.1^46^ and were aligned using Bowtie version 1.0.0^47^ to the following transcriptome databases: miRBase22 (miRNAs), a subset of RefSeq v90 (snoRNAs), and custom‐modified piRBase v2 (piRNA). Quantification was performed via SamTools^48^ python implementation, using a custom‐built bioinformatics architecture (Human Alignment Toolchain, HATCH, Quadrant Biosciences). To allow for efficient and meaningful alignment and quantification of RNAs from the piRBase v2 database, highly similar piRNA sequences were reduced using a hierarchical clustering approach and the resulting sequences were termed “wiRNAs.” The aligned reads were quantile normalized and each ncRNA feature was scaled (mean‐centered and divided by the feature standard deviation). The normalized ncRNA profiles for each sample were screened for sphericity using a principal component analysis prior to statistical analysis (Figure S2).

### 2.7 Statistical analysis

First, the training set (n = 430; mTBI = 201 and controls = 229) was used to identify ncRNA candidates for mTBI diagnosis. For each class of ncRNAs (ie, miRNAs, wiRNAs, and snoRNAs), a nonparametric Wilcoxon rank test with Bonferroni correction was used to identity features with significant differences between mTBI and control groups (false detection rate [FDR] < .05). Next, a partial least squares discriminant analysis (PLSDA) was used to visualize the ability of each ncRNA class to separate mTBI and control samples in two dimensions. Variable importance in projection was calculated for each ncRNA feature using the weighted sum of absolute regression coefficients. Then, a random forest analysis was performed (1000 trees and 10 features) to estimate the out‐of‐bounds error for each class of ncRNAs. Mean decrease in accuracy was estimated for each ncRNA feature. Finally, the top 10 features within each ncRNA class on Wilcoxon (Adj. *P*‐value), PLSDA (weighted coefficient sum), and Random Forest (mean decrease in accuracy) were pooled into a panel of ncRNA candidates with mTBI diagnostic potential. Duplicate features were removed from the candidate list. In total, 65 ncRNA candidates were iteratively assessed for diagnostic accuracy in the training set. Accuracy, defined by area under the receiver operating characteristic curve, was determined for both individual ncRNA features and ratios of features. To control for the potential effects of previous mTBI, sex, race, age, and underlying neuropsychological comorbidities (eg, anxiety, depression, chronic headache disorder, and ADHD) on ncRNA levels, these measures were scaled, log transformed, and incorporated as ratios with each ncRNA candidate. Missing values (1.8 % of all measures) were imputed with the singular value decomposition imputation method. Random forest was used to generate mTBI‐predictive models from sets of individual ncRNAs and ncRNA ratios. To avoid over‐modeling, and to produce a panel that could be rapidly assessed with qPCR in downstream applications, no more than 10 ncRNA features were allowed in each model. Area under the curve (AUC) in the training set was assessed with a 100‐fold Monte‐Carlo cross validation procedure: two thirds of the training set samples were used to evaluate feature importance and build a regression model for cross‐validation in the remaining one third of training set samples. The model with the highest AUC was chosen for external validation. Coefficients and features in the model were held constant and applied to the naïve test set (n = 108; mTBI = 50 and controls = 58). Sensitivity, specificity, positive predictive value (PPV), negative predictive value (NPV), and AUC were reported for both sets.

In the subset of training set participants assessed for post‐concussion symptoms, balance, neurocognition, and salivary ncRNA (n = 321; mTBI = 176 and control = 145), random forest was used to generate three models predicting mTBI status: (a) validated symptom scales (symptom severity and symptom burden on PCSS), (b) performance on four neurocognitive tasks, and (c) balance performance in eight stances. Age was included in each predictive model to control for its potential impacts on neurocognitive and balance performance, as well as symptom reporting. Model accuracy was assessed across the entire subset (n = 321) using 100‐fold cross‐validated (CV) AUC, as above. To mirror current clinical guidelines, a predictive model combining standardized symptom scores on the PCSS with neurocognitive measures was generated, and compared to combined models of PCSS with ncRNA levels, and PCSS with balance scores. Finally, to assess the maximum diagnostic accuracy yielded by combining all four approaches, random forest was used to generate a model combining PCSS scores, neurocognitive performance, balance, and ncRNA levels. To explore potential biases within each predictive model, medical and demographic features were compared between misclassified and correctly classified participants with a two‐tailed student's *t* test. Selection of ncRNA features and predictive model generation was performed with Metaboanalyst v4.0 online software.^49^

A post hoc analysis using Power Analysis and Sample Size Software (PASS version 15.0.4; NCSS, LLC, Kaysville, UT) determined that the sample size used in the training set provided >99% power to detect a difference between the null AUC (AUC = .70, indicative of acceptable clinical utility) and the alternative hypothesis (AUC = .85, estimated from our previously published research).^37^ A one‐sided *z*‐test was used with an alpha level set at .05 for continuous data with equal variances and binomial outcomes. The validation cohort achieved 87.6% power to differentiate the ncRNA model performance (AUC = .83) from the null hypothesis value (AUC = .70).

### 2.8 Physiologic relevance

To assess the physiologic relevance of the ncRNA features that were predictive of mTBI status, we used a three‐step approach. First, we assessed whether ncRNA levels trended back toward control levels over time, using 679 longitudinal samples collected from the mTBI participants. Nonparametric ANOVA was used to assess levels of the nine ncRNAs used in the diagnostic algorithm across five time points: <72 h (n = 129), 4‐7 days (n = 120), 8‐14 days (n = 190), 15‐30 days (n = 105), and 31‐60 days postinjury (n = 135). One sample per participant was used in each time point. Second, relationships between the nine ncRNAs of interest and individual symptoms (reported subjectively on the PCSS and measured objectively through neurocognitive and balance testing) were assessed with a Pearson correlation analysis. Third, high confidence gene targets (Targetscan Context Score < –0.5, *P*‐value < .01) for the miRNAs of interest were identified in DIANA miRPATH v3.0 online software.^50^ Overrepresentation of Kyoto Encyclopedia of Genes and Genomes (KEGG) pathways by these target genes was determined using a Fishers Exact Test with Bonferroni correction. For snoRNAs of interest, chromosome location, base pair size, orthologues, and proximal protein‐coding genes were provided using NCBI Entrez Gene. Diseases associated with the proximal gene were identified in Genecards.

## 3 RESULTS

### 3.1 Participant characteristics

On average, participants were 18 (±6) years of age (Table 1). The majority (331/538, 61%) were male and White race (335/538, 61%). There was no significant difference (*P* > .05) between the mTBI group and the control group in age, sex, or race for both the training and test sets. Self‐reported rates of anxiety (24/538, 4%), depression (20/538, 4%), ADHD (36/538, 7%), and chronic headache (25/538, 5%) were low. There were no significant differences between groups in anxiety or ADHD in both training and test sets. The mTBI group had higher rates of chronic headache in the training set (*P* = .022) and higher rates of depression in the test set (*P* = .029). Factors that could potentially impact saliva ncRNA content, such as average time of saliva collection (14:00 ± 4:00 h), mean body mass index (24 ± 7 kg/m^2^), rates of dietary restrictions (38/538, 7%), and recent exercise (77/538, 14%) did not differ significantly among mTBI and control groups in either the training or test set. The two groups had similar rates of prior lifetime concussions (141/538, 26%). There were 45 participants in the control group with OI (15%), and 20 sustained head impacts during exercise (mean hits‐to‐head = 8; range =1‐50).

**TABLE 1:** Participant medical and demographic characteristics

	All participants (n = 538)			Train set mTBI (n = 201)			Train set CTRL (n = 239)			*P*‐value	Test set mTBI (n = 50)			Test set CTRL (n = 58)			
	COUNT	AVG/PCT	SD	COUNT	AVG/PCT	SD	COUNT	AVG/PCT	SD		COUNT	AVG/PCT	SD	COUNT	AVG/PCT	SD	*P*‐value
Demographics																	
Age (years)	0	18.3	5.8	0	17.9	7.3		18.9	3.9	.09	0	17.1	7.1		18.4	4.16	.28
Sex (%M)	331	0.61		114	0.57		150	0.64		.05	29	0.58		38	0.66		.43
Race (White)	335	0.62		99	0.49		172	0.74		.74	26	0.52		38	0.66		.5
Race (Black)	48	0.09		11	0.05		25	0.11		.46	3	0.06		9	0.16		.29
Race (Asian)	6	0.01		1	0		5	0.02		.32	0	0		0	0		NA
Hispanic	22	0.04		4	0.02		13	0.06		.78	2	0.04		3	0.05		.66
Medical characteristics																	
Anxiety	24	0.04		6	0.03		14	0.06		.14	1	0.02		3	0.05		.39
Depression	20	0.04		6	0.03		10	0.04		.47	4	0.08		0	0		.03
Chronic headache	25	0.05		17	0.08		4	0.02		.02	4	0.08		0	0		.08
ADHD	36	0.07		10	0.05		19	0.08		.19	4	0.08		3	0.05		.55
Factors influencing saliva RNA																	
Collection time (24h clock)		14:00	3.7		14:00	3.6		14:00	3.9	.96		15:00	3.1		14:00	3.7	.5
Body mass index (kg/m^2^)		24.4	7.5		24.9	10.6		24	4.2	.23		23.8	5.9		24.9	5.7	.37
Dietary restrictions	38	0.07		14	0.07		17	0.07		.93	1	0.02		6	0.1		.09
Recent exercise	77	0.14		26	0.13		28	0.12		.83	13	0.26		10	0.17		.27
Injury history																	
History of concussion	141	0.26		59	0.29		58	0.25		.24	12	0.24		12	0.21		.59
Orthopedic injury	46	0.08					35	0.15						10	0.17		

### 3.2 Characteristics of mTBIs and related symptoms

On average, mTBI participants were enrolled 4 (± 4) days postinjury. The most common mechanism of injury was sport‐related (185/251, 74%), with football (59/251, 32%) and soccer (39/185, 21%) being the most common (Table 2). Falls (33/251, 13%), motor vehicle accidents (9/251, 4%), and “other” injury mechanisms (24/251, 10%) accounted for the remainder of mTBIs. Among mTBI participants, 73 of 251 (29%) reported retro‐ or antero‐grade memory loss, and 40 of 251 (16%) reported loss of consciousness immediately following the injury. There were 33 participants (16%) that reported postconcussive symptoms at 4 weeks beyond the time of injury. The most commonly reported symptoms among mTBI participants at the time of enrollment were “headache” (166/251, 66%), “don't feel right” (144/251, 57%), “pressure in head” (141/251, 56%), “difficulty concentrating” (136/251, 54%), and “fatigue” (134/251, 53%). The mTBI participants endorsed significantly greater severity (FDR < .05; 7‐point Likert scale) for each of the 22 symptoms when compared with control participants. Total symptom severity (sum of Likert scores for 22 symptoms) was also greater in the mTBI group (29 ± 26) than in the control group (4 ± 9; *P* < .001). The number of symptoms among mTBI participants (11/22 symptoms, ±7) was greater than for control participants (2/22 symptoms, ± 4; *P* < .001).

**TABLE 2:** Injury characteristics and mTBI symptoms

	All participants			All mTBI participants			All CTRL participants			*P*‐value	Train set mTBI			Train set CTRL			*P*‐value	Test set mTBI			Test set CTRL			
	COUNT	AVG/PCT	SD	COUNT	AVG/PCT	SD	COUNT	AVG/PCT	SD		COUNT	AVG/PCT	SD	COUNT	AVG/PCT	SD		COUNT	AVG/PCT	SD	COUNT	AVG/PCT	SD	*P*‐value
Days postinjury					4.3	3.5						4.3	3.57						4	3.3				
Injury mechanism																								
Sport				185	0.74						144	0.72						41	0.82					
MVC				9	0.04						9	0.04						0	0					
Fall				33	0.13						30	0.15						3	0.06					
Other				24	0.1						18	0.09						6	0.12					
Immediate symptoms																								
Memory loss following head injury				73	0.56						61	0.3						12	0.24					
Loss of consciousness at time of injury				40	0.34						33	0.16						7	0.14					
Persistent postconcussion symptoms				37	0.15						33	0.16						4	0.08					
Postconcussion symptom scale																								
Headache	158	1.4	1.8	166	2.4	1.8	20	0.2	0.7	2.6 × 10^–41^	130	2.3	1.8	15	0.2	0.8	1.2 × 10^–30^	36	2.7	1.6	5	0.2	0.4	6.2 × 10^–13^
Pressure in head	91	1.0	1.5	141	1.6	1.6	17	0.2	0.7	1.3E × 10^–25^	113	1.7	1.6	8	0.1	0.6	2.2 × 10^–22^	28	1.5	1.4	9	0.4	0.8	1.4 × 10^–4^
Neck pain	66	0.6	1.3	77	0.9	1.4	14	0.2	0.6	3.3 × 10^–10^	66	1.0	1.6	10	0.1	0.6	1.9 × 10^–9^	11	0.6	1.1	4	0.2	0.6	6.4 × 10^–2^
Nausea or vomiting	119	0.4	1.1	62	0.7	1.3	4	0.0	0.3	9.2 × 10^–11^	51	0.8	1.4	2	0.0	0.3	2.5 × 10^–9^	11	0.7	1.3	2	0.1	0.4	1.2 × 10^–2^
Dizziness	86	0.8	1.5	109	1.4	1.7	10	0.1	0.6	7.0 × 10^–18^	82	1.3	1.7	4	0.1	0.7	5.2 × 10^–13^	27	1.8	1.7	6	0.2	0.4	8.0 × 10^–7^
Blurred vision	107	0.5	1.2	78	0.8	1.3	8	0.1	0.6	5.1 × 10^–10^	60	0.8	1.4	7	0.1	0.7	1.7 × 10^–7^	18	0.9	1.4	1	0.0	0.2	4.8 × 10^–4^
Balance problems	147	0.6	1.1	90	0.9	1.2	17	0.1	0.5	7.6 × 10^–13^	72	0.9	1.3	13	0.1	0.5	4.3 × 10^–10^	18	1.0	1.3	4	0.1	0.3	4.4 × 10^–4^
Sensitivity to light	123	1.0	1.6	133	1.7	1.7	14	0.2	0.7	5.7 × 10^–25^	102	1.6	1.8	11	0.2	0.7	2.6 × 10^–18^	31	2.1	1.6	3	0.2	0.6	1.1 × 10^–8^
Sensitivity to noise	154	0.9	1.5	113	1.4	1.6	10	0.1	0.7	4.1 × 10^–20^	89	1.4	1.6	9	0.2	0.8	1.5 × 10^–14^	24	1.8	1.8	1	0.0	0.2	3.0 × 10^–7^
Feeling slowed down	116	1.0	1.5	132	1.6	1.6	22	0.2	0.5	9.3 × 10^–24^	111	1.6	1.7	15	0.2	0.5	1.2 × 10^–20^	21	1.4	1.5	7	0.3	0.6	1.9 × 10^–4^
Feeling like in a fog	162	0.7	1.4	104	1.3	1.6	12	0.1	0.4	2.1 × 10^–18^	84	1.3	1.7	9	0.1	0.4	1.1 × 10^–14^	20	1.5	1.8	3	0.1	0.3	5.0 × 10^–5^
Don't feel right	173	1.14	1.7	144	2.0	1.9	18	0.2	0.7	6.1 × 10^–28^	116	1.9	1.9	13	0.2	0.7	2.3 × 10^–21^	28	2.2	1.9	5	0.2	0.4	3.8 × 10^–8^
Difficulty concentrating	131	1.2	1.6	136	1.8	1.8	37	0.4	0.9	4.9 × 10^–19^	107	1.8	1.8	28	0.4	0.9	1.8 × 10^–15^	29	2.0	1.8	9	0.5	1.0	6.4 × 10^–5^
Difficulty remembering	174	0.8	1.4	110	1.3	1.5	21	0.2	0.7	5.0 × 10^–15^	90	1.3	1.5	17	0.2	0.7	5.7 × 10^–13^	20	1.3	1.7	4	0.3	0.9	2.0 × 10^–3^
Fatigue or low energy	96	1.2	1.7	134	1.8	1.9	40	0.4	0.9	7.2 × 10^–18^	110	1.9	1.9	30	0.4	0.9	1.4 × 10^–15^	24	1.7	1.8	10	0.5	1.0	1.4 × 10^–3^
Confusion	140	0.6	1.3	88	1.0	1.5	8	0.1	0.5	6.4 × 10^–14^	70	1.0	1.5	7	0.1	0.5	7.0 × 10^–11^	18	1.2	1.7	1	0.0	0.2	2.4 × 10^–4^
Drowsiness	86	1.0	1.6	118	1.6	1.8	22	0.2	0.7	2.6 × 10^–19^	96	1.6	1.8	17	0.2	0.7	1.7 × 10^–15^	22	1.8	2.0	5	0.2	0.6	3.8 × 10^–5^
More emotional	103	0.6	1.2	74	0.9	1.4	12	0.1	0.5	3.0 × 10^–10^	60	0.9	1.5	8	0.1	0.4	2.8 × 10^–9^	14	1.0	1.6	4	0.3	0.8	2.4 × 10^–2^
Irritability	63	0.7	1.4	82	1.1	1.6	21	0.2	0.6	2.6 × 10^–10^	65	1.0	1.7	17	0.2	0.5	1.2 × 10^–8^	17	1.2	1.6	4	0.3	0.9	6.6 × 10^–3^
Sadness	113	0.4	1.0	49	0.6	1.2	14	0.1	0.5	2.0 × 10^–5^	41	0.6	1.3	11	0.1	0.6	9.5 × 10^–5^	8	0.4	1.0	3	0.1	0.3	8.8 × 10^–2^
Nervous or anxious	115	0.7	1.3	81	0.9	1.4	32	0.4	0.9	1.0 × 10^–5^	66	0.9	1.5	25	0.4	1.0	9.1 × 10^–5^	15	0.9	1.4	7	0.4	0.8	4.5 × 10^–2^
Trouble falling asleep	284	0.7	1.4	74	1.0	1.6	41	0.4	0.9	1.3 × 10^–4^	63	1.1	1.7	30	0.4	0.9	7.5 × 10^–5^	11	0.7	1.3	11	0.6	1.0	7.0 × 10^–1^
Symptom burden		7.1	7.0		11.0	6.9		2.4	3.9	8.9 × 10^–42^		10.9	6.7		2.2	3.8	4.7 × 10^–34^		11.6	6.1		3.2	4.0	3.4 × 10^–9^
Symptom severity		17.5	23.4		28.8	25.5		4.1	9.1	1.1 × 10^–28^		28.5	26.3		4.0	9.6	2.8 × 10^–22^		30.4	22.9		4.9	6.8	3.9E‐08

### 3.3 Balance and neurocognitive assessment

Balance and cognitive testing were completed in a subset of mTBI (n = 179) and control (n = 147) participants using the ClearEdge Toolkit (Table S1). The magnitude of the difference between the groups was dependent on which balance and cognitive test was administered. The mTBI group displayed significantly (*P* < .05) greater body sway during TLEO (d = 0.57), TLEC (d = 0.53), TSEO (d = 0.46), TSEC (d = 0.48), and TLEOFP (d = 0.45) tasks compared with the control group. There were no significant differences (*P* > .05) in body sway between the two groups on TLECFP, TSEOFP, and TSECFP tasks. The mTBI group displayed significantly (*P* < .05) lower scores on all four neurocognitive assessments, including SRT1 (d = 1.1), SRT2 (d = 1.1), PRT (d = 0.97), and GNG (d = 1.1) testing.

### 3.4 Salivary RNA assessment

Among the 538 saliva samples assessed with high‐throughput RNA sequencing, the mean quality score was 34.6 (±0.4; range: 32.1‐35.1) and the average read count was 6.9 × 10^7^ (±4.1 × 10^7^; range: 7.9 × 10^6^ to 2.5 × 10^8^). After filtering out ncRNA features with <10 raw read counts in >90% of samples, there were 264 miRNAs, 4603 wiRNAs, and 176 Refseq RNAs (including lncRNAs and snoRNAs) remaining. The most common features in each ncRNA class were miR‐27b‐3p (present in 100%, mean raw read count: 32 619), RNA5‐8SN3 (present in 100% of samples, mean raw read count: 11 334), and wiRNA_2 (present in 100% of samples, mean raw read count: 538 703).

**FIGURE 1:**
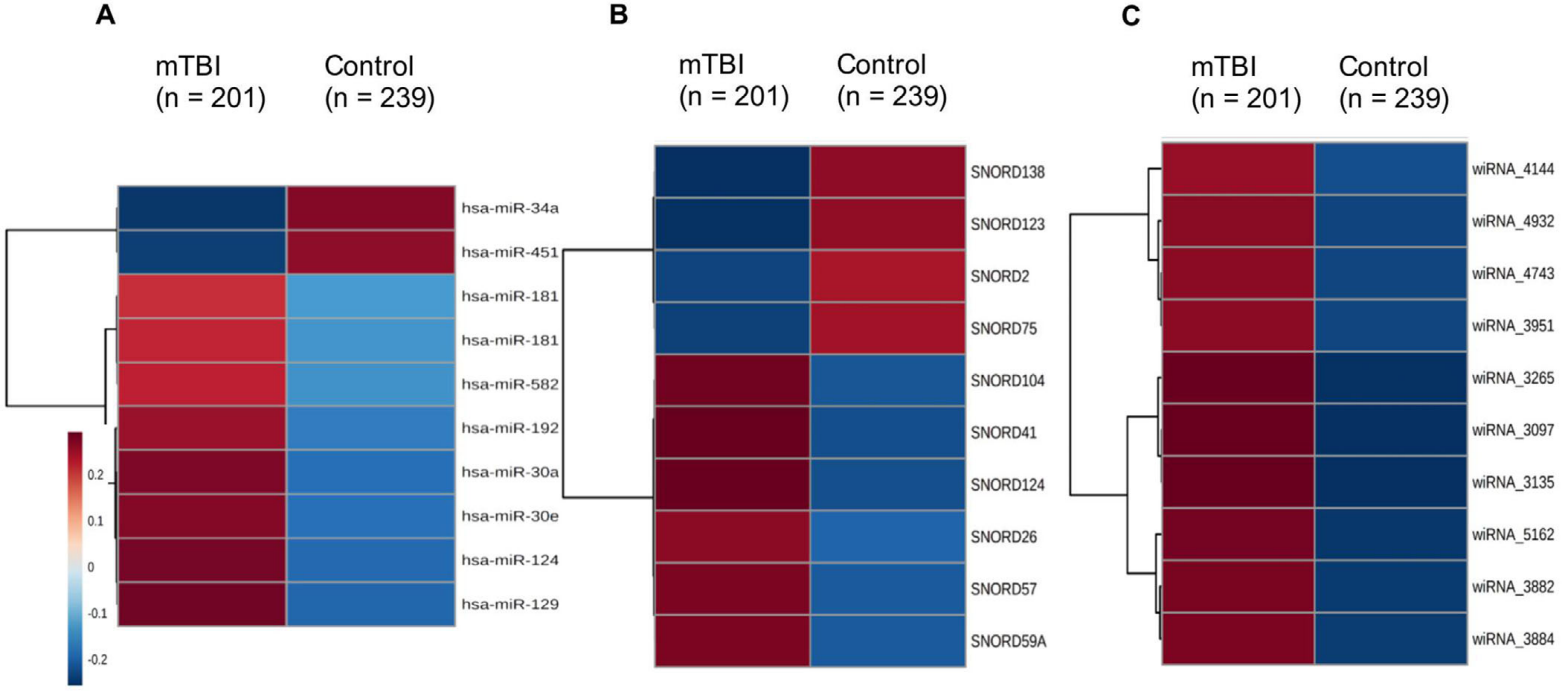
Noncoding RNAs with the largest differences between mTBI and control participants. The hierarchically clustered heatmaps display the 10 miRNAs (A), snoRNAs (B), and wiRNAs (C) with the largest differences between mTBI (n = 201) and control participants (n = 229) in the training set on Wilcoxon testing. Saliva levels of each noncoding RNA feature are denoted with red (high expression) or blue (low expression). Color scales indicate quantile‐normalized expression levels

There were 28 miRNAs, 21 Refseq RNAs, and 1378 wiRNAs with significant (FDR < .05) differences between mTBI and control groups on Wilcoxon testing (Table S2). There were 16 of 28 (57%) miRNAs, 12 of 21 (57%) Refseq RNAs, and 675 of 1378 (49%) wiRNAs upregulated in the mTBI group. The 10 ncRNA features in each class with the most significant between‐group differences are displayed in Figure 1A–C. Separate two‐dimensional PLSDAs employing each class of ncRNA were used to visualize their ability to differentiate mTBI and control groups. No class of ncRNAs was able to achieve full separation of mTBI and control groups on PLSDA (Figure S3A‐C). Coefficient scores estimating the importance of each individual ncRNA feature in sample projection were used to identify the top 10 RNA candidates within each category (Table S2). Random forest, employing 1000 trees and 10 features, was used to estimate the error rate for each class of ncRNAs for determining mTBI status (Figure S4A‐C). The 10 features within each ncRNA category that provided the largest mean decrease in accuracy on random forest were identified (Table S2). Finally, the top 10 ncRNAs within each category (miRNA, Refseq RNA, and wiRNA) from Wilcoxon, PLSDA, and random forest analyses were concatenated into a list of candidate features for mTBI diagnosis (Table 3). After removing duplicate features, there were 65 ncRNA features, including 22 miRNAs, 17 RefSeq RNAs, and 26 wiRNAs remaining.

**TABLE 3:** Noncoding RNA candidates for mTBI predictive models

miRNAs	snoRNAs	wiRNAs
hsa‐miR‐34a‐5p	SNORD138	wiRNA_3097
hsa‐miR‐30a‐3p	SNORD41	wiRNA_3135
hsa‐miR‐30e‐3p	SNORD2	wiRNA_4743
hsa‐miR‐1246	SNORD123	wiRNA_3265
hsa‐miR‐181c‐5p	SNORD57	wiRNA_3882
hsa‐miR‐192‐5p	SNORD104	wiRNA_3884
hsa‐miR‐4510	SNORD75	wiRNA_3951
hsa‐miR‐1290	SNORD59A	wiRNA_4932
hsa‐miR‐181a‐5p	SNORD124	wiRNA_5162
hsa‐miR‐582‐3p	SNORD26	wiRNA_4144
hsa‐miR‐12136	SNORA7B	wiRNA_4506
hsa‐miR‐944	SNORA7A	wiRNA_141
hsa‐let‐7e‐5p	SNORD88C	wiRNA_2569
hsa‐miR‐744‐5p	SNORD48	wiRNA_4844
hsa‐miR‐27a‐5p	SNORD20	wiRNA_12
hsa‐miR‐183‐5p	SNORD42A	wiRNA_4244
hsa‐miR‐708‐5p	SNORA109	wiRNA_2048
hsa‐miR‐1180‐3p		wiRNA_2447
hsa‐miR‐25‐3p		wiRNA_4384
hsa‐miR‐3074‐5p		wiRNA_4185
hsa‐miR‐3614‐5p		wiRNA_4023
hsa‐miR‐378a‐5p		wiRNA_125
		wiRNA_2188
		wiRNA_4024
		wiRNA_3833
		wiRNA_1520

### 3.5 Diagnostic utility

**FIGURE 2:**
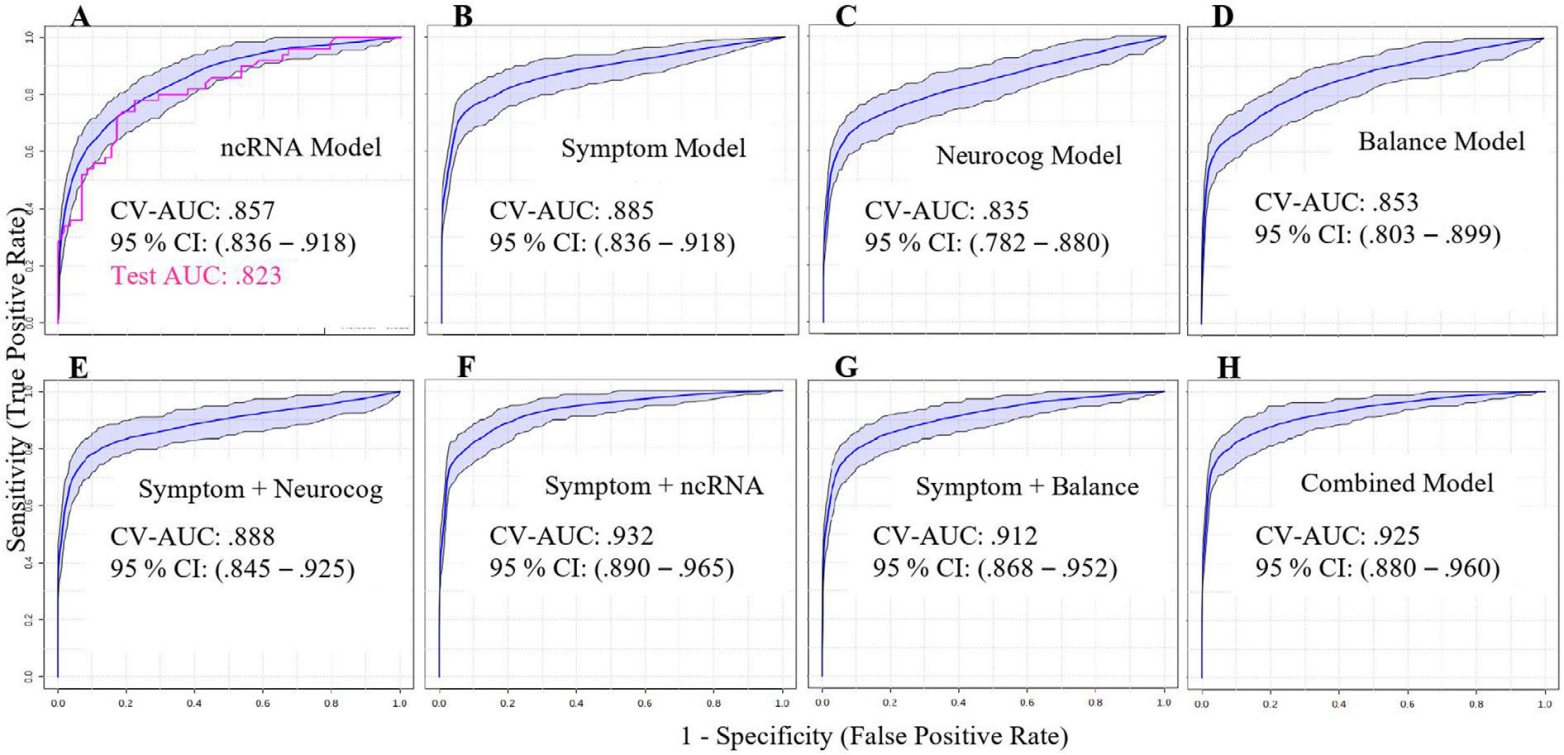
Diagnostic accuracy of saliva noncoding RNA, symptom scales, neurocognitive testing, and balance assessment for mTBI. A receiver operating characteristic (ROC) curve is displayed for a predictive model using seven ratios of nine non‐coding RNAs (ncRNAs) plus participant age and chronic headache history to differentiate individuals with mTBI from peers (A). Accuracy, determined by cross‐validated area under the curve (CV‐AUC), is shown for both training (blue; n = 430) and test sets (pink; n = 108). In a subset of participants (n = 321), cross‐validated accuracy of models employing symptom burden and symptom severity plus participant age (B), neurocognitive performance plus participant age (C), or balance performance plus participant age (D) were assessed. The combined predictive utility of symptom burden/severity and performance on four neurocognitive tests (E) was compared with symptom burden/severity and four saliva noncoding RNAs (F), symptom burden/severity and eight balance measures (G), and a model combining symptoms, neurocognition, balance, and saliva RNAs (H). Shaded areas denote the 95% confidence interval for CV‐AUC

A predictive model utilizing seven ratios, involving nine ncRNAs along with participant age and chronic headache status, differentiated mTBI and control participants with CV AUC of .857 (95% CI, .816‐.903) in the training set and an AUC of .823 in the test set (Figure 2A). The model correctly identified 190 of 251 (76%) mTBI participants and 232 of 287 (81%) control participants (PPV = 81%; NPV = 76%). In the subset of participants with symptom, neurocognitive, and balance data (n = 321), a model employing total number of symptoms and total symptom severity on the PCSS, along with participant age, was able to differentiate mTBI and control participants with CV AUC of .885 (95% CI, .836‐.918; Figure 2B). The PCSS model correctly identified 136 of 176 (77%) mTBI participants and 129 of 145 (89%) control participants (PPV = 89%; NPV = 76%). A model employing four neurocognitive measures along with participant age differentiated mTBI and control participants with CV AUC of .835 (95% CI, .782‐.880; Figure 2C). The model correctly identified 124 of 176 (70%) mTBI participants and 126 of 145 (87%) control participants (PPV = 87%; NPV = 75%). A model employing eight balance measures along with participant age differentiated mTBI and control participants with CV AUC of .853 (95% CI, .803‐.899; Figure 2D). The model correctly identified 126 of 176 (72%) mTBI participants and 127 of 145 (88%) control participants (PPV = 88%; NPV = 76%). Per clinical guidelines for mTBI assessment,^16^ we examined the combined ability of validated symptom scales and computerized neurocognitive testing to identify mTBI within our cohort. A predictive model employing symptom severity, symptom burden, performance on four neurocognitive measures, and age differentiated mTBI and control participants with CV AUC of .888 (95% CI, .845‐.925; Figure 2E). The model correctly identified 142 of 175 (81%) mTBI participants and 131 of 145 (90%) control participants (PPV = 90%; NPV= 81%). In comparison, a model combining symptom severity and symptom burden with levels of four ncRNAs and participant age differentiated mTBI and control participants with CV AUC of .932 (95% CI, .890‐.965; Figure 2F). The model correctly identified 146 of 176 (83%) mTBI participants and 129 of 145 (89%) control participants (PPV = 89%; NPV = 83%). A model combining symptom severity and symptom burden with eight balance tests and participant age differentiated mTBI and control participants with CV AUC of .912 (95% CI, .868‐.952; Figure 2G). The model correctly identified 140 of 176 (80%) mTBI participants and 128 of 145 (88%) control participants (PPV = 88%; NPV = 80%). Finally, a model combining all features (symptom severity, symptom burden, levels of four ncRNAs, performance on eight balance tests, and four neurocognitive tests, along with participant age) differentiated mTBI and control participants with CV AUC of .925 (95% CI, .880‐.960; Figure 2H). The model correctly identified 143 of 176 (81%) mTBI participants and 133 of 145 (92%) control participants (PPV = 92%; NPV = 81%). Components of each model can be found in Table S3.

### 3.6 Misclassification analysis

A two‐tailed student's *t*‐test was used to compare characteristics of correctly and incorrectly classified participants and identify potential biases within each model (Table S4). The ncRNA model was more likely (*P* < .05) to correctly classify participants with a history of previous concussion (*P* = .045), anxiety (*P* = .041), White race (*P* = .017), or OI (controls only, *P* = .037). Incorrect classification was more likely for participants with higher symptom severity (*P* = .0093), particularly those with high levels of headache symptoms (*P* = .014). However, the ncRNA model displayed no difference (*P* > .05) in classification accuracy for participants with other commonly reported symptoms, including “balance problems,” “fatigue,” or “difficulty concentrating.” The 37 mTBI participants who reported persistent symptoms 30 days postinjury were classified with similar accuracy as mTBI participants with symptom resolution by day 30 (*P* = .80). There was no difference between correctly and incorrectly classified participants in mTBI status, recent exercise, time since injury (mTBI group only), sex, age, body mass index, depression, or ADHD. For the model based on validated symptom scores, correct classification was more frequent than misclassification for participants with recent exercise (*P* = .015), history of previous concussion (*P* = .045), White race (*P* = .0025), and prolonged symptoms (*P* = .037). Incorrect classification was more likely for participants with mTBI (*P* = .00020), younger participants (*P* = .0019), and participants with ADHD (*P* = .047). There were no significant differences (*P* > .05) between correctly and incorrectly classified participants with respect to time since injury (mTBI group), body mass index, anxiety, or depression. The model combining ncRNA and validated symptom scores displayed no difference between correctly and incorrectly classified participants in mTBI status, recent exercise, time since injury (mTBI group), sex, history of previous concussion, body mass index, anxiety, depression, or ADHD. Correct classification was more likely for participants with younger age (*P* = .0079), White race (*P* = .0004), and prolonged symptoms (*P* = .039). The model was more accurate for participants reporting high levels of headache (*P* = .0003), balance problems (*P* = .013), difficulty concentrating (*P* = .0003), or fatigue (*P* = .0007), as well as those with higher symptom burden (*P* = .0013). Misclassification characteristics for balance and neurocognitive models are also displayed in Table S4.

### 3.7 Longitudinal trends for miRNAs that predict mTBI status

**FIGURE 3:**
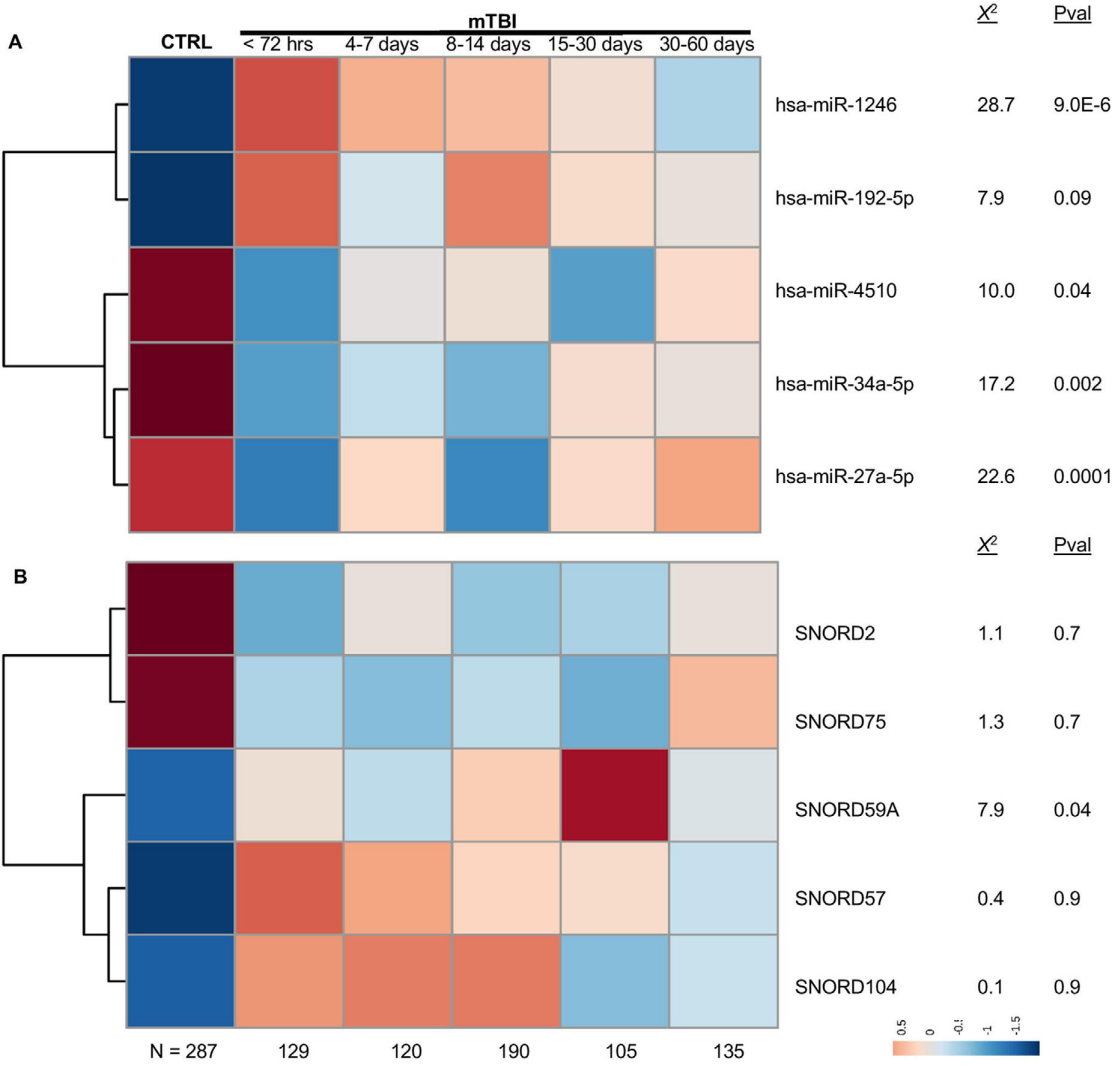
Longitudinal expression of diagnostic noncoding RNAs following mTBI. The heatmaps display salivary levels of five micro‐ribonucleic acids (miRNAs; A) and five small nucleolar RNAs (snoRNAs; B) used in the noncoding RNA (ncRNA) predictive model or the combined symptom/ncRNA model. Levels of each ncRNA are displayed for control participants (CTRL; N = 287) at enrollment, as well as mTBI participants (N = 251) sampled across 0‐3 days (n = 129), 4‐7 days (n = 120), 8‐14 days (n = 190), 15‐30 days (n = 105), and 30‐60 days (n = 135) postinjury. For each ncRNA, differences across postinjury time points were assessed with nonparametric analysis of variance (*χ* ^2^ and *P*‐values shown). Hierarchical clustering demonstrates relationships between individual ncRNA features. Blue denotes low expression and red denotes high expression. Color scales indicate quantile‐normalized expression levels

Nonparametric ANOVA was used to assess if the ncRNAs used to predict mTBI status displayed longitudinal trends toward a “control baseline” level in the 60 days postinjury. There was a significant difference (*P* < .05) in levels of four of five miRNAs across the four time points (Figure 3A). Only one of five snoRNAs displayed significant differences across the four time points (Figure 3B).

### 3.8 Relationships between ncRNAs and mTBI symptoms

**FIGURE 4:**
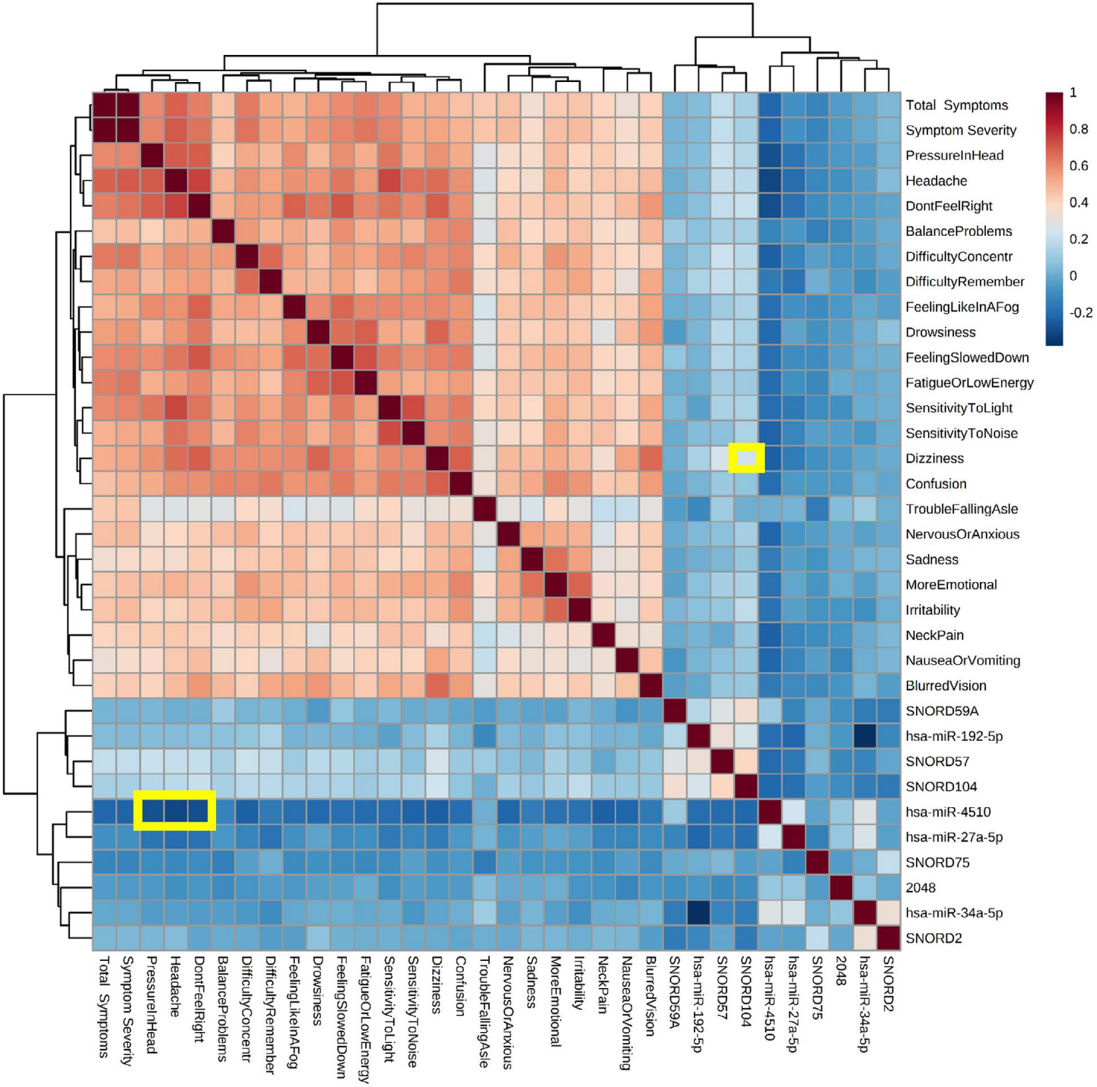
Associations between diagnostic noncoding RNAs and subjective symptom reports. The heatmap displays Pearson associations among 10 “diagnostic” salivary noncoding RNAs (ncRNAs), and 22 mTBI symptoms, as well as symptom burden and symptom severity. Saliva ncRNA was measured with high‐throughput sequencing and symptoms were self‐reported on the Post‐Concussion Symptom Scale by 321 participants (mTBI = 176; control = 145) at the time of enrollment. Color scales denote strength of association, where blue represents negative Pearson *R*‐values and red represents positive Pearson *R*‐values. Significant associations ([R] > .25; FDR < 0.001) between ncRNA features and specific mTBI symptoms are highlighted in yellow. Hierarchical clustering determined feature order

A Pearson correlation analysis was used to identify significant (FDR < .01) relationships between ncRNAs used in the predictive algorithm and mTBI symptoms (measured by PCSS, neurocognitive testing, and balance assessment) for 321 individuals. There were three ncRNAs associated with symptom severity (miR‐4510: *R* = –.22 and FDR = 3.4 × 10^–6^; SNORD57: *R* = .19 and FDR = 5.3 × 10^–4^; SNORD104: *R* = .13 and FDR = .008). Levels of miR‐4510 were most strongly associated with severity of “Headache,” whereas levels of SNORD104 and SNORD57 were most strongly associated with “Dizziness” (Figure 4). There were no ncRNAs from the predictive algorithm that were associated with performance scores on neurocognitive or balance testing (Figure S6A and S6B).

### 3.9 Physiologic relevance of predictive ncRNAs

The putative gene targets for the four miRNAs used in the predictive algorithm (miR‐34a‐5p, miR‐4510, miR‐27a‐5p, and miR‐192‐5p) were interrogated in DIANA miRPath v3 (Table 4). These four miRNAs demonstrated 259 high confidence messenger RNA targets, overrepresenting four physiologic pathways: synaptic vesicle cycle (FDR = 3.0 × 10^–5^, four genes, and two miRNAs), GABAergic synapse (FDR = .0026, three genes, and two miRNAs), transforming growth factor‐beta signaling pathway (FDR = .0026, four genes, and three miRNAs), and SNARE interactions in vesicular transport (FDR = .0056, four genes, and two miRNAs). The five snoRNAs used in the predictive algorithm arose from five separate chromosomes, and ranged from 60 to 75 base pairs in length. Protein coding genes proximal to these snoRNAs were implicated in several neurologic conditions, including Wernicke‐Korsakoff Syndrome (Tex2/SNORD104), Spinocerebellar Ataxia (Nop56/SNORD57), and Parkinson Disease (EIF4A2/SNORD2; Table S5).

**TABLE 4:** Putative physiologic targets of predictive microRNAs

KEGG pathway	*P*‐value	#genes	Genes	#miRNAs	miRNAs
Synaptic vesicle cycle	3.0 × 10^–5^	4	ATP6V1G2, ATP6V1B2, SYT1, VAMP2	2	miR‐34a‐5p, miR‐4510
GABAergic synapse	2.6 × 10^–3^	3	GABRG2, GABRA3, GLS2	2	miR‐4510, miR‐34a‐5p
TGF‐beta signaling pathway	2.6 × 10^–3^	4	THBS1, SMAD3, E2F5, LTBP1	3	miR‐27a‐5p, miR‐4510, miR‐34a‐5p
SNARE interactions in vesicular transport	5.6 × 10^–3^	4	GOSR1, VTI1B, VTI1A, VAMP2	2	miR‐34a‐5p, miR‐4510

## 4 DISCUSSION

This study identifies a set of ncRNA biomarkers in saliva that differentiate individuals with mTBI from peers without mTBI in both training (n = 430) and naive test sets (n = 108). In a subset of participants assessed with computerized neurocognitive testing, objective balance measures, and standardized symptom scales (n = 321), the ncRNA model displays similar accuracy for identifying mTBI status as these more traditional approaches.^16^ Importantly, the ncRNA model was not biased by recent exercise, time since injury (mTBI group), sex, history of previous concussion, body mass index, or underlying neuropsychologic conditions.

A model combining levels of four ncRNAs with subjective symptom reports yields comparable accuracy (AUC = .932) to that achieved with symptom reports and four neurocognitive measures (AUC = .888), or symptom reports and eight balance measures (AUC = .912). We note that none of these algorithms received the benefit of clinical acumen and injury history, which may explain why their diagnostic accuracy lags published rates.^51^ Unfortunately, the development of mTBI biomarkers depends upon comparison to subjective assessments on which the original mTBI diagnosis is based. This “circular comparison” can make it difficult to determine the true accuracy of any emerging technology. Here, we use a control group consisting of individuals with anxiety, depression, ADHD, OI, and exercise‐related fatigue to mimic many of the functional symptoms observed after mTBI. Although the majority of these control participants reported no mTBI symptoms, their mean symptom severity score (4) likely increased misclassification rates for mTBI participants with symptoms severity scores between one and four.

Current clinical guidelines recommend that healthcare providers use age‐appropriate symptom scales with or without computerized neurocognitive testing to diagnose mTBI, and specifically suggest that blood biomarkers only be used in research settings.^16^ The present results indicate that saliva ncRNA biomarkers yield similar diagnostic accuracy to a limited neurocognitive battery and provide additive value when combined with an algorithm relying solely on symptom scores (unaided by injury history). However, adding neurocognitive measures to a symptom/ncRNA model had minimal impact on accuracy. Thus, the additional time required to administer and interpret neurocognitive tests may not provide added benefit when combined with saliva ncRNA testing. This may be because neurocognitive testing and subjective symptom reports capture parallel information (ie, fatigue, concentration, and memory), whereas ncRNA may represent a more direct measure of biologic changes with additive value. Because biological changes do not provide information about the clinical manifestation of mTBI, it is crucial that such measures be used only as an adjunct to symptom reports and functional assessments.

In the present study, ncRNAs were measured with RNA sequencing technology, which requires >24 h to return results. However, we have previously shown that ncRNAs measured with a multiplex assay (≤4 h) yield comparable results.^39^ Emerging technology may soon provide the ability to measure ncRNAs field‐side using a portable device within 1 h.^52^, ^53^, ^54^ Until that time, balance and neurocognitive testing remain more expedient adjuncts. However, these assessments usually require an experienced healthcare provider, and many youth or high school competitions/practices do not have an experienced provider present. In comparison, minimal medical training is required to collect a saliva sample using the nucleic acid stabilizing kits employed here. Collection is completed in 10 s (without gagging the patient), samples are stabile at room temperature (for up to 3 months), and they can be transported to a lab without the biohazard regulations required for blood. Unlike neurocognitive testing, saliva ncRNA levels cannot be manipulated by individuals seeking to delay/expedite return to work or school.^17^, ^18^, ^19^

The scientific principle underlying our approach is that ncRNAs are packaged into vesicles and released into saliva by cranial nerves.^34^ The ncRNA content of salivary vesicles may change in order to regulate neuroinflammation and synaptic repair following mTBI. Indeed, functional analysis of the four miRNAs within the mTBI predictive model displays enrichment for genes involved in the synaptic vesicle cycle, SNARE interactions in vesicular transport, and GABAergic synapse. Numerous studies in animal models have identified alterations in GABA signaling networks following TBI,^55^, ^56^ and GABA imbalances have been implicated in specific symptomology following injury.^57^, ^58^ Transforming growth factor‐beta is also implicated as a physiologic mechanism underlying mTBI,^59^, ^60^ and the candidate ncRNAs target this pathway as well.

To our knowledge, this study is approximately five times larger than any previous study of ncRNA expression in mTBI.^33^ Additional strengths include the use of training and naïve test sets to validate the ncRNA diagnostic algorithm, and the recruitment of a control group with comparable age, sex, ethnicity, neuropsychologic history, and rates of previous concussion. Inclusion of individuals with recent exercise, OI, and subconcussive head impacts within the control group serves to increase the scientific rigor. Recruitment of participants from 11 different sites and multiple clinic settings (ie, ED, sports medicine clinics, urgent care, and outpatient primary care clinics) also promotes generalizability. Finally, direct comparison to current standard‐of‐care assessments provides initial evidence for the clinical utility of ncRNA biomarkers.

There are, however, several limitations to this study. No baseline cognitive or symptom data were available for participants, despite that fact that baseline testing is known to improve the accuracy of these measures.^61^, ^62^ Similarly, baseline levels of ncRNAs were not obtained, though reverse baseline measurements suggest preinjury saliva testing might also improve the accuracy of this technique. Although we attempted to match numerous medical and demographic characteristics across mTBI and control participants, complete matching was not possible for all categories, and rates of chronic headache disorder differed across groups. In addition, rates of neuropsychologic comorbidities (ie, ADHD, anxiety, and depression) are slightly lower in this cohort than in the general population,^63^ which may affect generalizability of the findings. Although the ncRNA predictive model was built and validated in a cohort of 538 individuals, only 321 of these individuals completed a full battery of neurocognitive, balance, and symptom assessments. Some participants (63/538; 12%) were unable/unwilling to complete the full battery of tests, some sites (3/11) could not administer the full battery within their clinic workflow, and individuals with primary language other than English were excluded because the neurocognitive battery required English fluency. Thus, this subcohort may include an element of selection bias. This also highlights some of the shortcomings involved with time‐consuming functional measures. We note that identification of mTBI participants in this study was reliant on physical and cognitive symptom assessments. Thus, the ncRNAs identified here may not improve sensitivity for mTBI identification. Future studies assessing ncRNA levels among asymptomatic individuals immediately following head impacts could determine if these molecules have the ability to increase diagnostic sensitivity.

Of the four miRNAs used in our ncRNA predictive algorithm, three (miR‐34a‐5p, miR‐192‐5p, and miR‐27a‐5p) were identified in previous studies of miRNA expression in individuals with TBI.^38^, ^62^, ^64^, ^65^ One miRNA (miR‐4510) has not been identified previously, and to our knowledge no previous mTBI study has interrogated snoRNAs or wiRNAs. Differences between our findings and existing literature may arise because of differences in (a) severity of brain injury; (b) participant age; (c) RNA quantification methodology; (d) sample sizes; and (e) biofluids. Even our own pilot studies of miRNA expression used an expectoration technique (rather than swab) to collect saliva, and involved younger participants.^37^

In conclusion, this study demonstrates that saliva ncRNA levels may be used as an objective measure to identify mTBI status in concert with currently available clinical tools. In the present cohort, this biologic measure has similar diagnostic accuracy to neurocognitive or balance testing, and displays additive value with standardized symptom assessment. External validation of this ncRNA model would provide additional evidence that biomarker testing deserves further consideration within clinical guidelines for mTBI diagnosis. Prognostic application of this technology may provide even greater clinical benefit—nearly 25% of individuals with mTBI suffer from prolonged symptoms, yet few accurate tools exist to predict the course of recovery. Given that saliva ncRNA measurement is objective, noninvasive, and does not require expert administration or interpretation, such a measure could represent a significant advance in standard of care for individuals with mTBI.

## CONFLICT OF INTERESTS

SDH is a paid consultant for Quadrant Biosciences. SDH and FAM are scientific advisory board members for Quadrant Biosciences and are named as co‐inventors on intellectual property related to saliva RNA biomarkers in concussion that are patented by The Penn State College of Medicine and The SUNY Upstate Research Foundation and licensed to Quadrant Biosciences. SDV, GF, and AR are paid employees of Quadrant Biosciences. CN is member of scientific advisory board and has equity interest in Quadrant Bioscience Inc. Material has been reviewed by the Walter Reed Army Institute of Research. There is no objection to its presentation and/or publication. The opinions or assertions contained herein are the private views of the author, and are not to be construed as official, or as reflecting true views of the Department of the Army or the Department of Defense. The investigators have adhered to the policies for protection of human subjects as prescribed in AR 70‐25. The other authors have no conflicts of interest to declare.

5

## Supporting information

Supporting information

## Data Availability

All FASTQ files used to generate the RNA sequencing dataset are the property of Quadrant Biosciences. Requests for data sharing will be reviewed and considered on a case‐by‐case basis.
