# Supplementary material for: Diagnosing mild traumatic brain injury using saliva RNA compared to cognitive and balance testing
Source: Clin Transl Med. 2020 Oct 4;10(6):e197. doi: 10.1002/ctm2.197 (PMC7533415; doi:10.1002/ctm2.197)
Supplement: Supplementary file 1 — Supporting information [file CTM2-10-e197-s001.docx]

**Table S1. Balance and neurocognitive performance (n = 326).**

|  |  | **All Participants** | | **mTBI Participants (n = 179)** | | **Control Participants (n = 147)** | | **P-value** |
| --- | --- | --- | --- | --- | --- | --- | --- | --- |
|  |  | AVG/PCT | STDEV | AVG/PCT | STDEV | AVG/PCT | STDEV |  |
| Balance | TLEO | 82.7 | 8.5 | 81.1 | 10.3 | 84.8 | 2.9 | 8.0E-05 |
|  | TLEC | 82.5 | 7.4 | 81.0 | 8.7 | 84.3 | 3.8 | 5.7E-05 |
|  | TSEO | 82.2 | 8.7 | 80.7 | 10.4 | 84.0 | 4.2 | 5.1E-04 |
|  | TSEC | 78.9 | 10.5 | 76.8 | 11.5 | 81.5 | 7.8 | 6.5E-05 |
|  | TLEOFP | 83.4 | 11.0 | 81.5 | 12.7 | 85.8 | 6.5 | 4.0E-04 |
|  | TLECFP | 83.9 | 7.1 | 83.2 | 7.3 | 84.7 | 6.6 | 5.4E-02 |
|  | TSEOFP | 84.5 | 4.9 | 84.2 | 5.0 | 84.8 | 4.5 | 2.8E-01 |
|  | TSECFP | 84.3 | 5.0 | 84.1 | 5.2 | 84.6 | 4.7 | 4.1E-01 |
| Neurocognitive | SRT1 | 1721.0 | 35.4 | 156.9 | 36.0 | 191.2 | 24.0 | 1.5E-20 |
|  | PRT | 90.6 | 18.0 | 83.3 | 18.2 | 99.9 | 12.9 | 1.6E-18 |
|  | GNG | 111.5 | 18.9 | 104.4 | 19.7 | 120.4 | 13.1 | 1.4E-15 |
|  | SRT2 | 168.5 | 33.1 | 154.8 | 33.8 | 185.8 | 22.7 | 5.2E-19 |

**Table S2. Top non-coding RNA biomarkers for mTBI (within miRNA, snoRNA, and wiRNA categories) on Wilcoxon, PLSDA and Random Forest analyses.**

|  | Wilcoxon Rank Test | | | | | | PLSDA | Random Forest |
| --- | --- | --- | --- | --- | --- | --- | --- | --- |
|  | Fold Change | log2(FC) | V | p.value | -LOG10(p) | FDR | PLSDA weighted regression coefficient | RF Mean Decrease Accuracy |
| **miRNA** |  |  |  |  |  |  |  |  |
| hsa-miR-4510 | 0.39 | -1.36 | 18228 | 2.0E-04 | 3.71 | 1.0E-02 | 100.00 | 3.9E-03 |
| hsa-miR-34a-5p | 0.61 | -0.71 | 16702 | 9.2E-07 | 6.04 | 0.0E+00 | 53.79 | 2.8E-03 |
| hsa-miR-744-5p | 0.79 | -0.34 | 19750 | 1.1E-02 | 1.95 | 8.0E-02 | 49.15 | 1.7E-03 |
| hsa-miR-192-5p | 1.79 | 0.84 | 27970 | 1.2E-04 | 3.93 | 1.0E-02 | 22.57 | 1.5E-03 |
| hsa-miR-25-3p | 1.71 | 0.77 | 27018 | 1.8E-03 | 2.73 | 3.0E-02 | 30.78 | 1.2E-03 |
| hsa-miR-30e-3p | 1.88 | 0.91 | 28704 | 9.7E-06 | 5.02 | 0.0E+00 | 24.69 | 1.2E-03 |
| hsa-miR-30a-3p | 1.92 | 0.94 | 28732 | 8.7E-06 | 5.06 | 0.0E+00 | 25.93 | 1.2E-03 |
| hsa-miR-3074-5p | 0.72 | -0.47 | 19388 | 4.8E-03 | 2.32 | 5.0E-02 | 33.49 | 9.2E-04 |
| hsa-miR-3614-5p | 0.96 | -0.05 | 25901 | 2.5E-02 | 1.61 | 1.1E-01 | 7.69 | 8.1E-04 |
| hsa-miR-378a-5p | 0.57 | -0.81 | 18556 | 5.3E-04 | 3.28 | 1.0E-02 | 24.97 | 8.0E-04 |
| hsa-miR-27a-5p | 0.41 | -1.27 | 18713 | 8.2E-04 | 3.09 | 2.0E-02 | 45.14 | 7.5E-04 |
| hsa-miR-181c-5p | 1.52 | 0.61 | 28040 | 9.3E-05 | 4.03 | 0.0E+00 | 5.49 | 7.0E-04 |
| hsa-miR-708-5p | 0.58 | -0.79 | 18594 | 5.9E-04 | 3.23 | 1.0E-02 | 41.58 | 6.1E-04 |
| hsa-miR-1246 | 3.82 | 1.93 | 28200 | 5.5E-05 | 4.26 | 0.0E+00 | 29.43 | 6.0E-04 |
| hsa-let-7e-5p | 0.64 | -0.64 | 19814 | 1.3E-02 | 1.89 | 8.0E-02 | 49.87 | 5.1E-04 |
| hsa-miR-944 | 1.51 | 0.59 | 26238 | 1.2E-02 | 1.91 | 8.0E-02 | 50.43 | 4.5E-04 |
| hsa-miR-1290 | 3.26 | 1.71 | 27810 | 1.9E-04 | 3.72 | 1.0E-02 | 37.21 | 2.5E-04 |
| hsa-miR-181a-5p | 1.45 | 0.54 | 27843 | 1.7E-04 | 3.76 | 1.0E-02 | 7.89 | 2.4E-04 |
| hsa-miR-582-3p | 1.60 | 0.67 | 27734 | 2.4E-04 | 3.62 | 1.0E-02 | 21.64 | 1.6E-04 |
| hsa-miR-183-5p | 1.00 | 0.01 | 23498 | 7.1E-01 | 0.15 | 8.1E-01 | 44.40 | 1.0E-04 |
| hsa-miR-1180-3p | 0.65 | -0.61 | 20917 | 1.0E-01 | 0.99 | 2.5E-01 | 41.23 | 2.4E-05 |
| hsa-miR-12136 | 0.56 | -0.83 | 19371 | 4.6E-03 | 2.34 | 5.0E-02 | 70.87 | -2.4E-05 |
| **snoRNA** |  |  |  |  |  |  |  |  |
| SNORA7B | 5.36 | 2.42 | 25614 | 4.3E-02 | 1.36 | 1.5E-01 | 100.00 | 4.0E-03 |
| SNORD138 | 0.61 | -0.72 | 17012 | 3.0E-06 | 5.52 | 0.0E+00 | 77.59 | 3.7E-03 |
| SNORD2 | 0.56 | -0.84 | 17510 | 1.9E-05 | 4.73 | 0.0E+00 | 63.44 | 3.6E-03 |
| SNORD59A | 1.98 | 0.99 | 27664 | 3.0E-04 | 3.52 | 1.0E-02 | 65.99 | 2.7E-03 |
| SNORA7A | 4.71 | 2.23 | 25886 | 2.6E-02 | 1.59 | 1.2E-01 | 98.71 | 2.6E-03 |
| SNORD20 | 1.82 | 0.86 | 26004 | 2.0E-02 | 1.70 | 1.0E-01 | 58.93 | 2.5E-03 |
| SNORD104 | 2.33 | 1.22 | 28069 | 8.5E-05 | 4.07 | 0.0E+00 | 61.52 | 2.0E-03 |
| SNORD42A | 0.86 | -0.21 | 19782 | 1.2E-02 | 1.92 | 7.0E-02 | 44.16 | 1.8E-03 |
| SNORD57 | 1.71 | 0.77 | 28126 | 7.0E-05 | 4.15 | 0.0E+00 | 64.29 | 1.7E-03 |
| SNORA109 | 0.88 | -0.19 | 19720 | 1.0E-02 | 1.98 | 7.0E-02 | 28.20 | 1.7E-03 |
| SNORD41 | 2.00 | 1.00 | 28674 | 1.1E-05 | 4.97 | 0.0E+00 | 84.87 | 1.6E-03 |
| SNORD123 | 0.57 | -0.80 | 17827 | 5.5E-05 | 4.26 | 0.0E+00 | 56.55 | 1.6E-03 |
| SNORD75 | 0.58 | -0.79 | 18088 | 1.3E-04 | 3.89 | 0.0E+00 | 85.90 | 1.5E-03 |
| SNORD26 | 2.14 | 1.10 | 27268 | 9.4E-04 | 3.03 | 2.0E-02 | 53.85 | 1.4E-03 |
| SNORD88C | 0.49 | -1.02 | 26160 | 1.4E-02 | 1.84 | 8.0E-02 | 65.09 | 1.2E-03 |
|  | Wilcoxon Rank Test | | | | | | PLSDA | Random Forest |
|  | Fold Change | log2(FC) | V | p.value | -LOG10(p) | FDR | PLSDA weighted regression coefficient | RF Mean Decrease Accuracy |
| SNORD124 | 2.47 | 1.30 | 27300 | 8.6E-04 | 3.07 | 2.0E-02 | 69.10 | 6.5E-04 |
| SNORD48 | 0.54 | -0.90 | 19331 | 4.2E-03 | 2.38 | 4.0E-02 | 64.45 | 1.0E-04 |
| **wiRNA** |  |  |  |  |  |  |  |  |
| wiRNA_3097 | 4.97 | 2.31 | 30721 | 2.1E-09 | 8.69 | 0.0E+00 | 84.53 | 5.4E-04 |
| wiRNA_4185 | 5.47 | 2.45 | 29858 | 1.0E-07 | 6.99 | 0.0E+00 | 80.00 | 4.5E-04 |
| wiRNA_4023 | 6.21 | 2.63 | 30102 | 3.5E-08 | 7.45 | 0.0E+00 | 77.28 | 4.0E-04 |
| wiRNA_125 | 1.86 | 0.90 | 29790 | 1.4E-07 | 6.86 | 0.0E+00 | 22.77 | 3.5E-04 |
| wiRNA_4932 | 6.57 | 2.72 | 30438 | 7.8E-09 | 8.11 | 0.0E+00 | 77.49 | 3.4E-04 |
| wiRNA_2188 | 2.44 | 1.29 | 28700 | 9.8E-06 | 5.01 | 0.0E+00 | 44.20 | 3.3E-04 |
| wiRNA_3951 | 2.78 | 1.47 | 30544 | 4.8E-09 | 8.32 | 0.0E+00 | 70.47 | 3.0E-04 |
| wiRNA_4024 | 6.17 | 2.63 | 30103 | 3.5E-08 | 7.45 | 0.0E+00 | 77.91 | 3.0E-04 |
| wiRNA_3833 | 3.41 | 1.77 | 29956 | 6.7E-08 | 7.17 | 0.0E+00 | 61.32 | 2.9E-04 |
| wiRNA_1520 | 0.66 | -0.59 | 17249 | 7.3E-06 | 5.13 | 0.0E+00 | 20.67 | 2.9E-04 |
| wiRNA_4743 | 6.77 | 2.76 | 30718 | 2.1E-09 | 8.68 | 0.0E+00 | 76.03 | 2.7E-04 |
| wiRNA_2048 | 0.46 | -1.12 | 20829 | 8.9E-02 | 1.05 | 1.6E-01 | 89.25 | 1.9E-04 |
| wiRNA_5162 | 12.82 | 3.68 | 30426 | 8.2E-09 | 8.09 | 0.0E+00 | 74.73 | 1.7E-04 |
| wiRNA_3884 | 6.18 | 2.63 | 30404 | 9.1E-09 | 8.04 | 0.0E+00 | 80.32 | 1.7E-04 |
| wiRNA_2569 | 0.55 | -0.86 | 17664 | 3.2E-05 | 4.50 | 0.0E+00 | 96.07 | 9.5E-05 |
| wiRNA_4844 | 1.76 | 0.82 | 28118 | 7.2E-05 | 4.14 | 0.0E+00 | 93.16 | 5.2E-05 |
| wiRNA_4506 | 1.64 | 0.72 | 25790 | 3.1E-02 | 1.51 | 8.0E-02 | 100.00 | 3.3E-05 |
| wiRNA_3882 | 6.23 | 2.64 | 30428 | 8.2E-09 | 8.09 | 0.0E+00 | 81.34 | 3.1E-05 |
| wiRNA_1417 | 1.03 | 0.04 | 21602 | 2.7E-01 | 0.57 | 3.7E-01 | 14.01 | 3.0E-05 |
| wiRNA_12 | 0.52 | -0.95 | 17188 | 5.9E-06 | 5.23 | 0.0E+00 | 93.04 | 2.2E-05 |
| wiRNA_4144 | 2.69 | 1.43 | 30264 | 1.7E-08 | 7.77 | 0.0E+00 | 66.80 | 8.2E-06 |
| wiRNA_2447 | 0.40 | -1.34 | 16308 | 1.8E-07 | 6.74 | 0.0E+00 | 88.77 | 6.8E-06 |
| wiRNA_4244 | 1.96 | 0.97 | 27202 | 1.1E-03 | 2.95 | 0.01 | 90.96 | -7.4E-05 |

**Table S3. Diagnostic Algorithms**

| **Model** | **Components** |
| --- | --- |
| ncRNA Model | Age, miR-34a-5p/SNORD57, miR-34a-5p/SNORD104, miR-4510/SNORD59A, chronic headache/miR-27a-5p, miR-34a-5p/miR-192-5p, miR-192-5p/SNORD2, SNORD57/SNORD75 |
| Symptom Model | Symptom Burden, Symptom Severity |
| Neurocognitive Model | SRT1, PRT, SRT2, GNG, Age |
| Balance Model | Age, TSEC, TSEC, TLEC, TLEO, TLEOFP, TLECFP, TSECFP, TSEOFP |
| Symptom + Neurocognitive Model | Symptom Burden, Symptom Severity, Age, SRT1, PRT, SRT2, GNG |
| Symptom + ncRNA Model | Age, Symptom Severity, Symptom Burden, miR-4510, miR-27a-5p, miR-1246, wiRNA_2048 |
| Symptom + Balance Model | Symptom Burden, Symptom Severity, Age, TSEC, TLEO, TLEC, TSEO, TLEOFP, TLECFP, TSECFP, TSEOFP |
| Combined Model | Symptom Burden, Symptom Severity, Age, PRT, SRT2, miR-4510, SRT1, GNG, miR-27a-5p, miR-1246, wiRNA_2048, TSEC, TLEO, TLEC, TSEO, TLEOFP, TLECFP, TSECFP, TSEOFP |

Note: external validation of these algorithms will require use of the ClearEdge Toolkit for assessment of balance and neurocognition. Algorithms employing ncRNA levels require exact replication of saliva collection and handling, RNA extraction and quantification procedures, and scaling/normalization steps reported in this manuscript. Abbreviations: simple reaction time (SRT1), procedural reaction time (PRT), go/no-go (GNG), repeat of the simple reaction time test (SRT2), two legs eyes open (TLEO), tandem stance eyes open (TSEO), two legs eyes closed (TLEC), tandem stance eyes closed (TSEC), two legs eyes open on a foam pad (TLEOFP), two legs eyes closed on a foam pad (TLECFP), tandem stance eyes open on a foam pad (TSEOFP), and tandem stance eyes closed on a foam pad (TSECFP).

**Table S4. Misclassification characteristics of non-coding RNA, symptom, balance, and neurocognition predictive models.**

|  | **ncRNA Model** | | | **Symptom Model** | | | **Neurocog Model** | | | **Balance Model** | | | **Symptom + Neurocog Model** | | | **Symptom + ncRNA Model** | | | **Symptom + Balance Model** | | | **Combined Model** | | |
| --- | --- | --- | --- | --- | --- | --- | --- | --- | --- | --- | --- | --- | --- | --- | --- | --- | --- | --- | --- | --- | --- | --- | --- | --- |
|  | Correct | Incorrect | Pval | Correct | Incorrect | Pval | Correct | Incorrect | Pval | Correct | Incorrect | Pval | Correct | Incorrect | Pval | Correct | Incorrect | Pval | Correct | Incorrect | Pval | Correct | Incorrect | Pval |
| mTBI status | 0.44 | 0.53 | 0.08 | 0.51 | 0.76 | 0 | 0.5 | 0.72 | 0 | 0.39 | 0.74 | 0 | 0.4 | 0.69 | 0.03 | 0.53 | 0.65 | 0.13 | 0.41 | 0.68 | 0.04 | 0.52 | 0.72 | 0.01 |
| Exercise | 0.14 | 0.14 | 0.98 | 0.19 | 0.06 | 0.02 | 0.19 | 0.09 | 0.04 | 0.34 | 0.1 | 0.07 | 0.31 | 0.14 | 0.61 | 0.18 | 0.11 | 0.24 | 0.29 | 0.17 | 0.97 | 0.18 | 0.11 | 0.24 |
| Days post-injury (mTBI group only) | 21.68 | 4.2 | 0.43 | 25.67 | 7.58 | 0.55 | 27.96 | 5.2 | 0.47 | 4.42 | 5.29 | 0.46 | 4.94 | 5.72 | 0.59 | 22.85 | 15.81 | 0.85 | 4.94 | 5.82 | 0.58 | 25.18 | 5.85 | 0.6 |
| Sex (%M) | 1.41 | 1.32 | 0.09 | 1.39 | 1.37 | 0.7 | 1.41 | 1.33 | 0.26 | 1.35 | 1.35 | 0.37 | 1.35 | 1.37 | 0.7 | 1.4 | 1.37 | 0.73 | 1.35 | 1.36 | 0.58 | 1.4 | 1.33 | 0.32 |
| Age (years) | 18.33 | 18.28 | 0.94 | 18.04 | 19.74 | 0 | 17.85 | 20.34 | 0 | 18.47 | 20.44 | 0 | 18.68 | 19.98 | 0 | 18.15 | 19.78 | 0.01 | 18.65 | 20.15 | 0 | 18.1 | 20.11 | 0 |
| Prior concussion | 0.29 | 0.21 | 0.05 | 0.28 | 0.16 | 0.05 | 0.27 | 0.25 | 0.75 | 0.19 | 0.21 | 0.24 | 0.18 | 0.16 | 0.09 | 0.28 | 0.15 | 0.07 | 0.17 | 0.19 | 0.19 | 0.27 | 0.2 | 0.27 |
| Body mass index | 24.2 | 24.93 | 0.32 | 24.26 | 25.97 | 0.15 | 24.18 | 25.98 | 0.12 | 24.94 | 25.7 | 0.2 | 24.87 | 26.52 | 0.08 | 24.26 | 26.42 | 0.11 | 24.73 | 26.45 | 0.08 | 24.23 | 26.6 | 0.08 |
| Anxiety | 0.06 | 0.02 | 0.04 | 0.02 | 0.02 | 0.72 | 0.02 | 0.03 | 0.64 | 0 | 0.04 | 0.19 | 0 | 0.02 | 0.93 | 0.03 | 0 | 0.27 | 0 | 0.02 | 0.86 | 0.03 | 0 | 0.27 |
| Depression | 0.04 | 0.04 | 0.91 | 0.02 | 0.05 | 0.11 | 0.02 | 0.01 | 0.65 | 0 | 0.01 | 0.61 | 0 | 0.02 | 0.94 | 0.02 | 0.02 | 0.99 | 0 | 0.02 | 0.87 | 0.02 | 0.02 | 0.99 |
| ADHD | 0.07 | 0.07 | 0.81 | 0.03 | 0.08 | 0.05 | 0.02 | 0.1 | 0 | 0.01 | 0.08 | 0.02 | 0.01 | 0.1 | 0.01 | 0.03 | 0.07 | 0.29 | 0.02 | 0.08 | 0.11 | 0.03 | 0.09 | 0.06 |
| Race (White) | 0.87 | 0.77 | 0.02 | 0.89 | 0.7 | 0 | 0.89 | 0.73 | 0.01 | 0.79 | 0.75 | 0.01 | 0.84 | 0.62 | 0 | 0.89 | 0.59 | 0 | 0.86 | 0.65 | 0 | 0.89 | 0.59 | 0 |
| Headache | 1.23 | 1.72 | 0.01 | 1.42 | 0.84 | 0.02 | 1.36 | 0.99 | 0.11 | 1.11 | 1.08 | 0.27 | 1.14 | 0.47 | 0 | 1.41 | 0.46 | 0 | 1.16 | 0.55 | 0 | 1.4 | 0.54 | 0 |
| Balance problems | 0.51 | 0.66 | 0.21 | 0.55 | 0.26 | 0.04 | 0.52 | 0.32 | 0.12 | 0.55 | 0.36 | 0.24 | 0.54 | 0.22 | 0.04 | 0.53 | 0.15 | 0.01 | 0.54 | 0.21 | 0.02 | 0.53 | 0.15 | 0.01 |
| Difficulty concentrating | 1.06 | 1.39 | 0.08 | 1.17 | 0.66 | 0.03 | 1.12 | 0.74 | 0.07 | 1.05 | 0.96 | 0.62 | 1.02 | 0.47 | 0 | 1.14 | 0.43 | 0 | 1.01 | 0.55 | 0.01 | 1.12 | 0.54 | 0.02 |
| Difficulty remembering | 0.68 | 1.06 | 0.01 | 0.79 | 0.37 | 0.03 | 0.74 | 0.49 | 0.15 | 0.64 | 0.51 | 0.18 | 0.62 | 0.22 | 0 | 0.76 | 0.24 | 0.01 | 0.6 | 0.28 | 0.01 | 0.75 | 0.33 | 0.03 |
| Fatigue or low energy | 1.09 | 1.43 | 0.08 | 1.24 | 0.53 | 0 | 1.21 | 0.68 | 0.02 | 0.9 | 0.83 | 0.13 | 0.94 | 0.33 | 0 | 1.22 | 0.35 | 0 | 0.94 | 0.36 | 0 | 1.21 | 0.41 | 0 |
| Symptom severity | 15.55 | 22.47 | 0.01 | 18.47 | 9.21 | 0.01 | 17.39 | 12.33 | 0.1 | 14.28 | 14.33 | 0.41 | 14.63 | 5.63 | 0 | 18.13 | 5.37 | 0 | 14.54 | 6.45 | 0 | 17.98 | 6.3 | 0 |
| Persistent post-concussion symptoms | 0.14 | 0.16 | 0.8 | 0.2 | 0.06 | 0.04 | 0.19 | 0.08 | 0.07 | 0.16 | 0.08 | 0.05 | 0.21 | 0.03 | 0.02 | 0.18 | 0.03 | 0.04 | 0.21 | 0.03 | 0.02 | 0.19 | 0.03 | 0.02 |
|  |  |  |  |  |  |  |  |  |  |  |  |  |  |  |  |  |  |  |  |  |  |  |  |  |
| "Incorrect" denotes average rate (percentage) of specified condition among incorrectly classified participants | | | | | | | | |  |  |  |  |  |  |  |  |  |  |  |  |  |  |  |  |
| "Correct" denotes average rate (percentage) of specified condition among correctly classified participants | | | | | | | | |  |  |  |  |  |  |  |  |  |  |  |  |  |  |  |  |

**Table S5. Characteristics of predictive snoRNAs.**

| **ID** | **Name** | **Location** | **Splice Variants** | **Orthologues** | **Size (bp)** | **Proximal Protein-Coding Gene** | **Disease Associations** |
| --- | --- | --- | --- | --- | --- | --- | --- |
| SNORD104 | small nucleolar RNA, C/D box 104 | Chromosome 17: 64,146,083-64,146,152 | 1 | 119 | 70 | TEX2 | Wernicke-Korsakoff Syndrome |
| SNORD59A | small nucleolar RNA, C/D box 59A | Chromosome 12: 56,645,027-56,645,101 | 1 | 249 | 75 | ATP5F-1B | Hydronephrosis |
| SNORD57 | small nucleolar RNA, C/D box 57 | Chromosome 20: 2,656,939-2,657,010 | 1 | 170 | 72 | NOP56 | Spinocerebellar Ataxia |
| SNORD75 | small nucleolar RNA, C/D box 75 | Chromosome 1:173,836,017-173,836,076 | 1 | 152 | 60 | SERPINC1 | Thrombosis |
| SNORD2 | small nucleolar RNA, C/D box 2 | Chromosome 3: 186,784,796-186,784,864 | 1 | 171 | 69 | EIF4A2 | Parkinson Disease |

**Fig. S1.**

**Enrolled Participants (n = 580)**

**Eligible Participants (n = 567)**

**Excluded (n = 25)**

**Reasons:** Control participants with age > 30 years, and absence of recent exercise, OI, and neuro-psychologic conditions excluded to match medical and demographic characteristics with mTBI group.

**Matched Participants (n = 542)**

**Eligible mTBI Participants (n = 251)**

**Eligible Control Participants (n = 291)**

**Valid Control Samples (n = 287)**

**Valid mTBI Samples (n = 251)**

**Excluded (n = 0)**

**Reasons:** The earliest sample after mTBI was used for diagnostic comparison (n = 251). 428 additional samples (679 total) were used for longitudinal analysis.

**mTBI training set (n = 201)**

**mTBI test set (n = 50)**

**Control test set (n = 58)**

**Control train set (n = 229)**

**Excluded (n = 4)**

**Reasons:** Aligned read counts < 5x10^5^

**Excluded (n = 13)**

**Reasons:** Sample collected > 14 days after mTBI (ineligible for mTBI group), and < 12 weeks after mTBI (ineligible for control group).

**Figure S1. Flow chart for participant enrollment and exclusion.** Of the 580 participants initially enrolled, 13 were excluded because enrollment occurred > 14 days post-mTBI. There were 25 controls excluded to balance age, and rates of recent exercise, orthopedic injury (OI), and neuropsychological conditions (i.e. ADHD, anxiety, depression) across groups. Samples from four controls were excluded due to low RNA read counts. Of the 679 saliva samples collected from mTBI participants, 251 (one per participant) was used for the diagnostic comparison. Remaining samples were binned into five time-points (one sample per participant, per time-point) for down-stream longitudinal analysis.

**Fig. S2.**


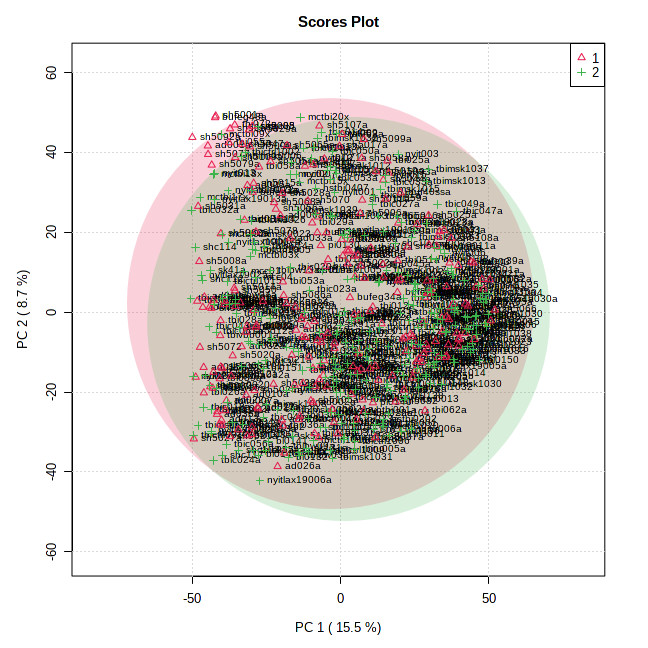


Principal Component 1 (15.5%)

Principal Component 2 (8.7%)

**Figure S2. Saliva non-coding RNA profiles demonstrate sphericity.** A principal components analysis was used to visualize sphericity of the non-coding RNA (ncRNA) data set, and ensure the absence of outliers resulting from RNA quantification, alignment, or normalization. The two-dimensional PCA accounts for 24.2 % of variance in the ncRNA data. Red Δ (1) represents mTBI participants, while green + (2) represents controls.

**Fig. S3.**


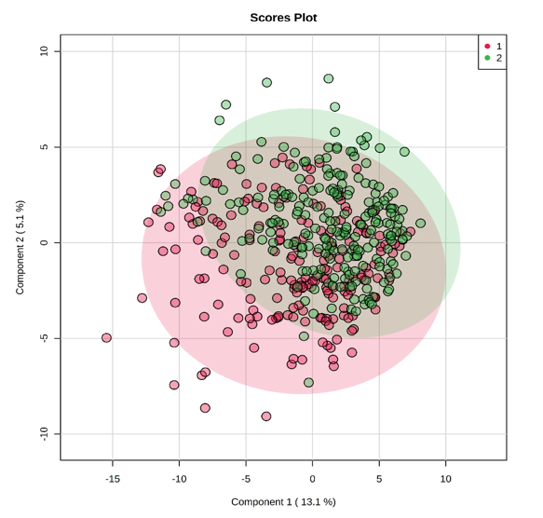


microRNAs

Component 2 (5.1 %)

Component 1 (13.1 %)


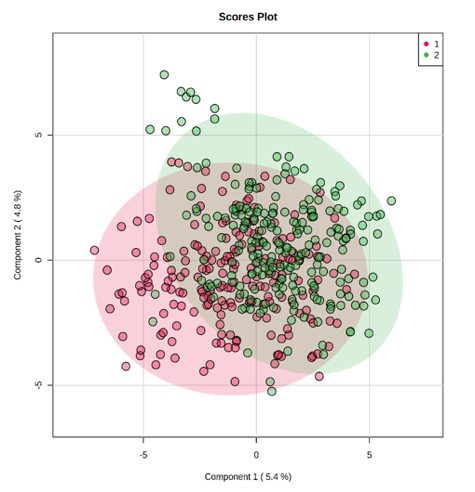


Component 2 (4.8 %)

Component 1 (5.4 %)

snoRNAs

**A**

**B**

**C**


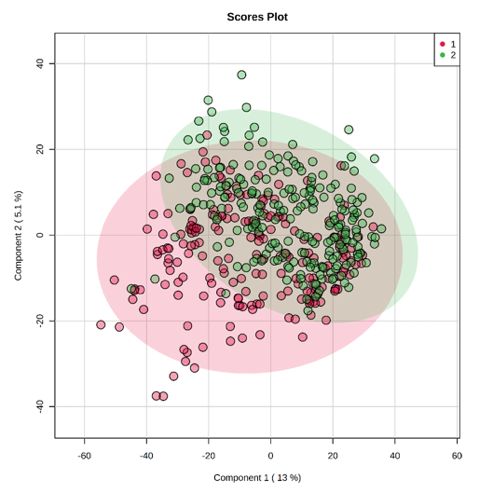


wiRNAs

Component 2 (5.1 %)

Component 1 (13.0 %)

mTBI

Control

mTBI

Control

mTBI

Control

**Figure S3. Partial least squares discriminant analysis (PLSDA) of non-coding RNA features.** A PLSDA was used to examine the ability of micro-ribonucleic acids (microRNAs; A), small nucleolar RNAs (snoRNAs; B), and piwi-interacting RNAs (wiRNAs; C) to differentiate mTBI (red; n = 201) and control (green; n = 239) participants in the training set. All three classes of non-coding features achieved partial separation of the groups, while accounting for 18.2 %, 10.2 %, and 18.1 % of variance in the data, respectively.

**Fig. S4.**


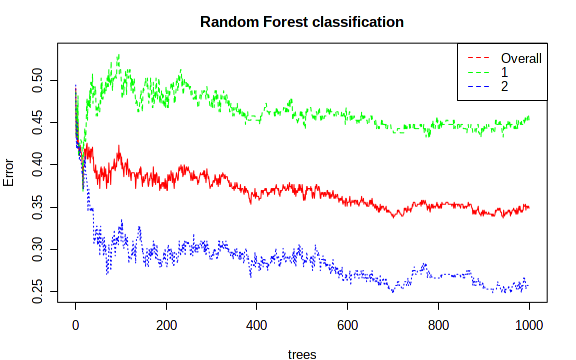

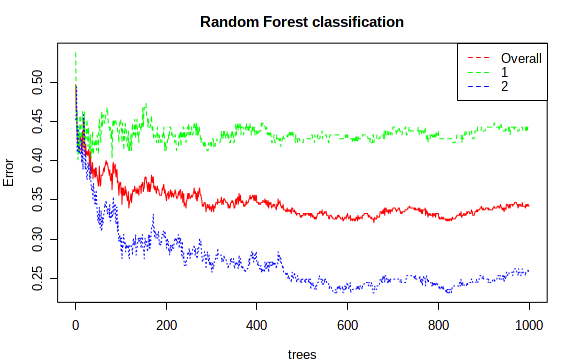

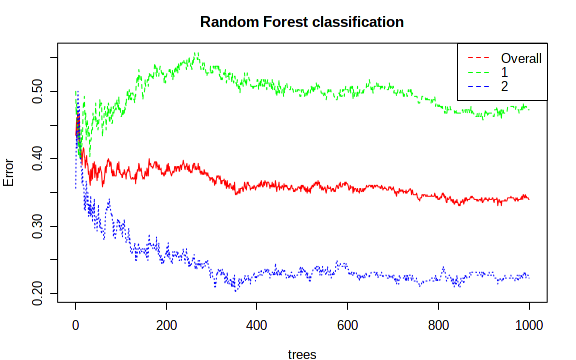


Trees

Error

**A**

**B**

**C**


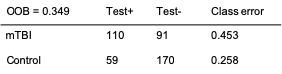

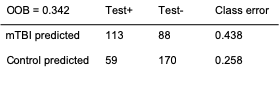

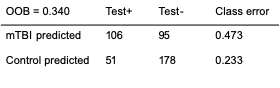


miRNA

snoRNA

wiRNA

**Figure S4. Random forest classification with non-coding RNA features.** A random forest approach, utilizing 1000 trees was applied to identify non-coding features with predictive utility for mTBI status. Out of bounds (OOB) and classification (class) error rates are shown for a miRNA model (A), snoRNA model (B), and wiRNA model (C), which each used 10 features.

**Fig. S5.**


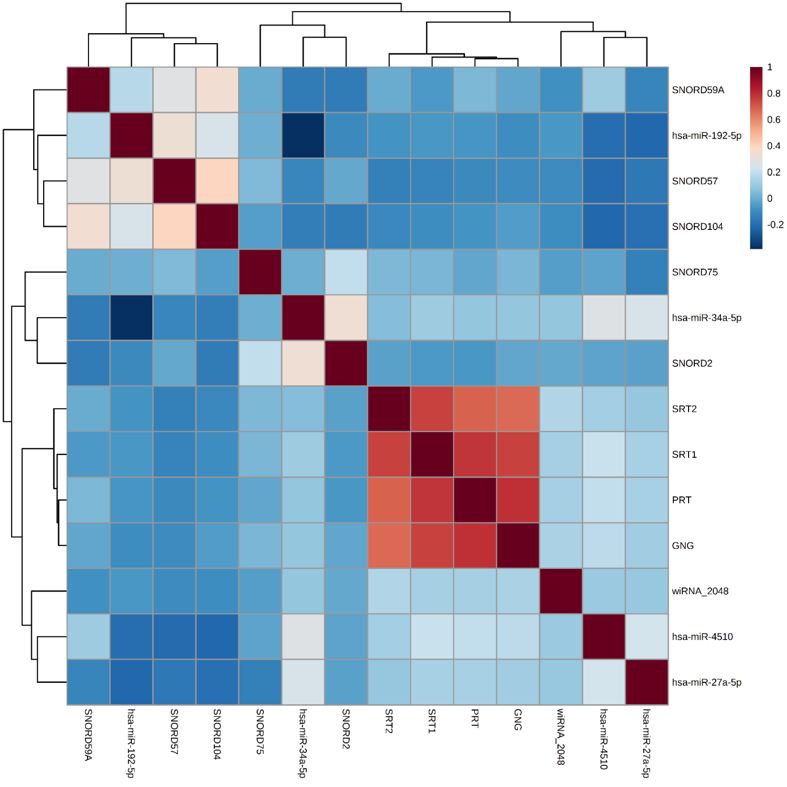


**A**


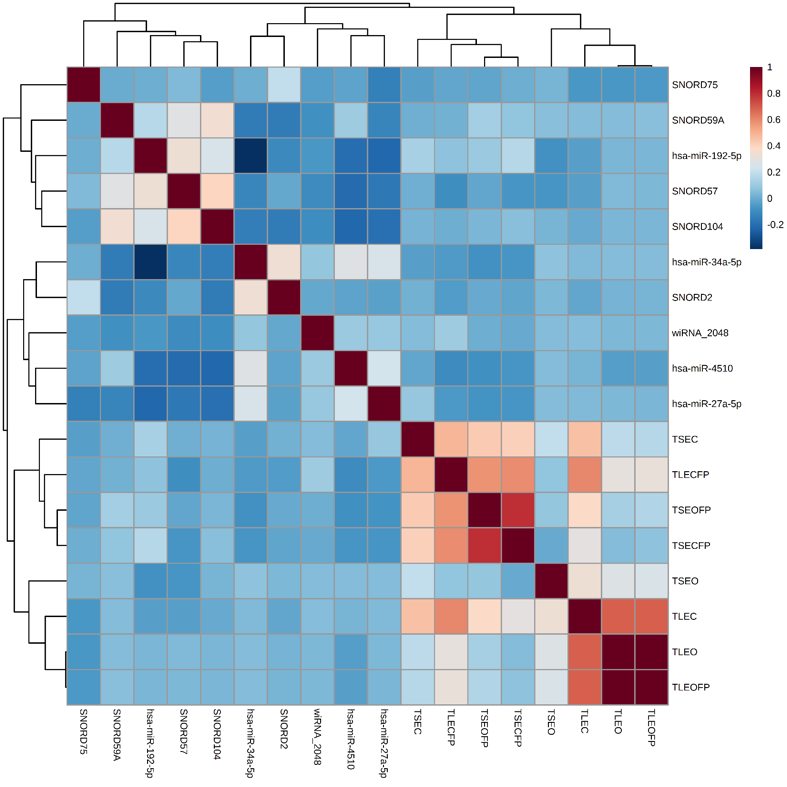


**B**


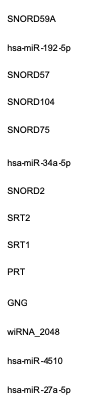

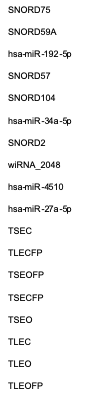


**Figure S5. Associations of diagnostic non-coding RNAs with functional clinical measures.** The heatmaps display associations between the non-coding RNAs (ncRNAs) used in the two predictive models (Figure 2A & 2F) and measures of neurocognition (A), or balance (B). There were no significant ([R] > 0.25, FDR < 0.001) associations between ncRNAs and clinical measures.
